# Assessment of hydrogeophysical delineation of groundwater zones using vertical electrical sounding (VES) in Jeypore Block, Koraput District, Odisha

**DOI:** 10.1038/s41598-026-59721-2

**Published:** 2026-07-27

**Authors:** Tanmoy Chatterjee, Surajit Munshi, Arunkumar Yadav, Biswajit Ghosh, Janmejay Sethy, Duryadhan Behera, Shreerup Goswami

**Affiliations:** 1 Department of Earth Sciences, Sambalpur University, Jyoti Vihar, Sambalpur, Burla, 768019 Odisha India; 2 Department of Civil Engineering, School of Engineering and Technology, Dhamma Dipa International Buddhist University, South Tripura, Sabroom, Tripura 799145 India; 3 Manipal Institute of Technology Bengaluru, Manipal Academy of Higher Education, Manipal, India; 4 Centre for Climate Change and Water Research, Suresh Gyan Vihar University, Mahal, Jagatpura, Jaipur, Rajasthan 302025 India; 5 Amity Institute Forestry and Wildlife, Amity University, Gautam Buddh Nagar, Sector 125, Noida, Uttar Pradesh 201301 India; 6 Department of Geology, Utkal University, Vani Vihar, Bhubaneswar, 751004 Odisha India

**Keywords:** Groundwater potential, Vertical Electrical Sounding (VES), Analytical Hierarchy Process (AHP), GIS-based multi-criteria analysis, Eastern Ghats Mobile Belt, Environmental sciences, Hydrology, Natural hazards, Solid Earth sciences

## Abstract

Groundwater occurrence in hard-rock terrains is complex and spatially heterogeneous, controlled by weathered and fractured zones with limited surface expression. This research integrates Vertical Electrical Sounding (VES) with remote sensing, GIS and Analytical Hierarchy Process (AHP) for groundwater potential mapping of Jeypore Block, Koraput District, Odisha. This area is underlain by Eastern Ghats Mobile Belt rocks such as khondalites, charnockites, and granite gneisses. Fifteen VES surveys using Schlumberger configuration were conducted across the study area, with electrode spreads up to 800 m. Resistivity data were interpreted using IPI2WIN software, producing one-dimensional resistivity models and Dar-Zarrouk parameters. Subsurface profiling revealed distinct lithological layers consisting of topsoil, laterite, sandstone, weathered/fractured granite, and compact bedrock. Layer thicknesses ranged from 0.6 to 20.4 m in the first layer to 99.5 m in the third layer. Iso-resistivity maps generated using Surfer-25 showed significant lateral heterogeneity. Low-resistivity zones (2.6–105.2 Ωm) indicated saturated formations, whereas high-resistivity values (> 150 Ωm) represented compact basement rocks. Curve-type analysis identified predominantly AAA-type curves, which indicate increasing resistivity with depth. HA-type curves observed at two locations suggested the presence of conductive, water-saturated layers favourable for aquifer development. VES-derived parameters were integrated with twelve geospatial thematic layers, including geology, geomorphology, soil, NDVI, lineament density, drainage density, rainfall, slope, physiography, land use, groundwater fluctuation, and hydrogeology, using the AHP framework. The consistency ratio of the model was 2.36%, indicating reliable thematic weighting. Validation via pumping tests and ROC analysis (AUC = 0.86) confirmed model reliability. This approach offers a scientifically robust framework for sustainable groundwater management in structurally complex hard-rock terrains.

**Supplementary Information:**

The online version contains supplementary material available at 10.1038/s41598-026-59721-2.

## Introduction

Access to clean water, reliable transportation, and energy forms the foundation of sustainable community development, with water being the most vital and universally required resource for life^1^. Due to rapid population growth and urbanization, the strain on both surface and groundwater systems has increased, prompting the need for advanced technologies to map and monitor water availability for sustainable management^2–7^. In this context, evaluating groundwater potential, particularly in hard rock and basement complex terrains, is essential for securing long-term water supply for domestic, agricultural, and industrial purposes (Adeyeye et al., 2018;^8–11^)

Groundwater occurrence and movement in hard rock terrains are largely influenced by geographic, geomorphological, structural, and climatic conditions^12–15^; Rachna, 2024). Although water may be naturally abundant, its accessibility, especially in urban and peri-urban settings, is hindered by contamination and the uneven distribution of groundwater stored in weathered and fractured basement rocks^1,16–20^.

The combination of satellite-based data with ground validation enables the detection of topographic, structural, and geomorphic features that indicate groundwater potential^21,22^. Geographic Information Systems (GIS) play a crucial role in managing and analyzing spatial hydrogeological data, enhancing the accuracy of groundwater assessments. In addition, techniques like Vertical Electrical Sounding (VES) have proven effective and affordable for identifying groundwater zones, especially in geologically complex or poorly studied regions^15,22,23^.

Groundwater is a vital freshwater source for billions worldwide, yet it faces escalating threats from over-extraction, pollution, and climate change, contributing to severe water crises in many regions^24^. In India’s Indo-Gangetic Plain, particularly Punjab, Haryana, and Rajasthan, groundwater levels are dropping by nearly one meter per year due to intensive agricultural use, resulting in desertification, lower crop productivity, and increased farmer distress^25^. The North China Plain, including Beijing and Hebei, is similarly affected by overuse for agriculture and industry, leading to land subsidence and persistent water shortages^26^. In Iran’s Central Plateau, unregulated groundwater extraction has pushed over 60% of aquifers into critical condition, causing urban land subsidence and extensive environmental harm^27^. Mexico City continues to experience severe ground sinking due to over-pumping, while Egypt’s Nile Delta faces saline intrusion that threatens freshwater supply and agriculture^28^. Human activities have now overtaken natural forces as the primary drivers of environmental change, placing unprecedented stress on ecosystems^29^. Climate change and groundwater depletion, largely driven by fossil fuel emissions and unsustainable water use, are key contributors to rising temperatures, sea level rise, and intensifying water scarcity^26^. Additionally, urban expansion, deforestation, and large-scale agriculture are accelerating ecosystem degradation, while overpopulation, poor infrastructure planning, and industrial pollution further erode environmental resilience^22,30^.

Modern techniques such as Vertical Electrical Sounding (VES), GIS, Remote Sensing, and Geoinformatics are widely used for studying environmental and geophysical changes due to their specific analytical strengths^31^. VES is particularly effective for subsurface investigations, offering detailed insights into groundwater distribution, soil layering, and potential contamination zones by measuring electrical resistivity at varying depths^32^. This method provides accurate, site-specific data critical for identifying aquifer characteristics and understanding underground geological conditions^33^. However, one key limitation of VES is its point-based nature, which restricts its ability to represent broader spatial patterns across large areas^34^. To address this, Geoinformatics integrates VES outputs with spatial technologies such as GIS and Remote Sensing, allowing for more comprehensive visualization and modeling of environmental variables^35^. GIS excels in analyzing surface-level patterns by combining multiple datasets to map land use, urban growth, and ecological changes^36^. Meanwhile, Remote Sensing provides satellite-based data for monitoring large-scale environmental dynamics like vegetation cover, erosion, and climate impacts^37^. Collectively, these geospatial tools offer a multidimensional approach that enhances environmental monitoring and supports sustainable natural resource management^38^.

Although Jeypore Block lies within an undulating physiographic setting, several low-lying areas of Koraput District experience seasonal groundwater shortages due to limited aquifer recharge^39^. However, despite the growing concern over groundwater availability in this region, systematic and location-specific empirical investigations focusing on groundwater recharge dynamics, seasonal variability, and local hydrogeological controls within Jeypore Block remain limited. Most existing studies have addressed groundwater conditions at broader regional scales, leaving a significant gap in detailed assessments that capture the spatial heterogeneity and localized recharge characteristics of this block^39–44^. Addressing this gap is therefore essential for improving the understanding of groundwater availability and for supporting sustainable groundwater management strategies in Jeypore Block and the surrounding parts of Koraput District.The region’s complex hard rock geology, characterized by groundwater storage in weathered and fractured zones with few surface indicators, poses major challenges for aquifer development^15,23,26,31,45,46^. Despite increasing water demand, existing studies have not adequately captured the spatial complexity of subsurface conditions in this geologically diverse terrain. To address the identified research gap, this study establishes clearly defined objectives focusing on groundwater exploration in Jeypore Block. The primary objectives are to evaluate subsurface hydrogeological conditions using Vertical Electrical Sounding (VES) and to integrate these geophysical observations with Remote Sensing and GIS-based spatial analysis for systematic delineation of groundwater potential zones. Through this integrated framework, the study aims to improve the scientific understanding of groundwater occurrence in hard-rock terrains and provide a reliable basis for sustainable groundwater resource planning and management in the region.

The adoption of the 1D VES technique is justified by its cost-effectiveness, operational simplicity, and suitability for preliminary aquifer characterization in hard-rock terrains where rugged topography, limited accessibility, and resource constraints restrict the application of advanced surveys such as Electrical Resistivity Tomography and a scientifically grounded framework to guide sustainable groundwater management in the Jeypore Block.

While the integration of Vertical Electrical Sounding (VES) with GIS-based Analytical Hierarchy Process (AHP) has been widely applied for groundwater potential mapping, the present study introduces important refinements at both methodological and regional scales. First, the study explicitly integrates Dar–Zarrouk parameters (e.g., longitudinal conductance and transverse resistance) into the groundwater potential zonation scheme. This provides a quantitative link between the geoelectrical and aquifer hydraulic properties, which has not been taken advantage of in most GIS-based studies of this type. Second, the study is situated within the Eastern Ghats Mobile Belt (EGMB), a geologically complex high-grade metamorphic terrain characterized by khondalites, charnockites, and granite gneisses, where groundwater occurrence is primarily controlled by secondary porosity associated with weathering and fracturing. The application of an integrated VES–GIS–AHP approach in such a tectonically evolved and heterogeneous setting provides new insights into groundwater occurrence mechanisms that differ from sedimentary or less deformed crystalline terrains. Finally, unlike many regional-scale studies, the present work incorporates field-based validation through pumping test data and well yield analysis, thereby strengthening the reliability of geophysical interpretations and enhancing the practical applicability of the groundwater potential model for sustainable resource planning.

## Materials and methods

### Study area

**Fig. 1:**
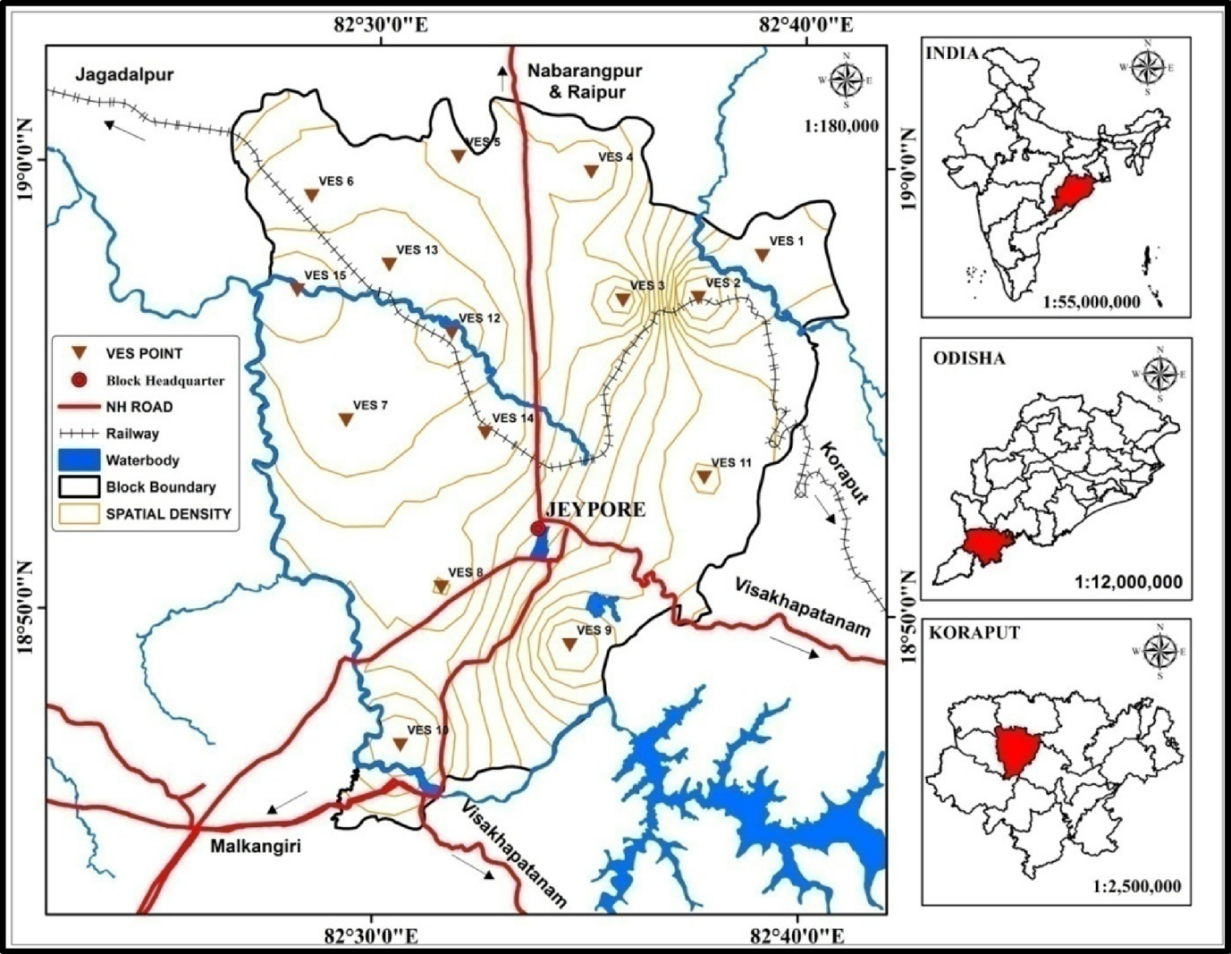
Spatial distribution of Vertical Electrical Sounding (VES) points and groundwater prospect zones around Jeypore.

Jeypore Block, located in the south-western part of Odisha within Koraput District, spans an area of approximately 456 square kilometres between latitudes 18°29’11” N to 19°01’45” N and longitudes 82°26’31” E to 82°41’06” E, with an average elevation of 693 m above mean sea level Chatterjee et al^39,42,43^, Chatterjee et al.^40^,). Bordered by Barigumma, Dasamanthpur, Koraput, Boipariguda, Kundra, and Katpad, it serves as a key administrative and ecological zone. Geologically, the block is part of the Eastern Ghats Mobile Belt (EGMB), composed of Archean to Proterozoic high-grade metamorphic rocks such as khondalites, charnockites, granite gneisses, and migmatites^44^. These formations have undergone multiple deformation and metamorphic events, resulting in a highly heterogeneous subsurface and the location identified by the spatial density of VES point (Fig. 1). Groundwater in this region is predominantly stored within weathered layers and fracture networks of the granite-gneiss complex, with structural features like faults and joints enhancing secondary porosity and permeability^47^. The underlying soils are largely red loamy to lateritic, moderately fertile, and support mostly rain-fed agriculture. With annual rainfall between 1300 and 1600 mm from the southwest monsoon, land use in the block includes 64% agricultural land, 23% forest cover, and 13% fallow or non-cultivable land^47^. The study area in Koraput District receives an average annual rainfall of approximately 1600 mm, mostly during the June–September monsoon. Still, shallow hard-rock aquifers composed of weathered and fractured crystalline rocks provide limited storage, leading to seasonal water scarcity in many villages (CGWB, 2024). To address this, the study integrates Vertical Electrical Sounding (VES) with GIS analysis to delineate groundwater potential zones, improving understanding of subsurface heterogeneity, guiding borewell targeting, and supporting sustainable groundwater management in rain-fed, topographically complex terrains. The study area exhibits complex hard-rock geology, varied geomorphological conditions, and uneven groundwater occurrence, which present significant challenges for groundwater investigation. To address these conditions, VES surveys were strategically conducted across different geological and geomorphological settings and integrated with multiple thematic layers to improve spatial interpretation. The reliability of the results was further supported by pumping-test validation and ROC analysis (AUC = 0.86), indicating good predictive performance of the groundwater potential model. Nevertheless, future studies incorporating denser geophysical surveys and multi-season observations would further enhance the understanding of subsurface heterogeneity and groundwater dynamics in the region.

## Methods

### Data collection

**Fig. 2:**
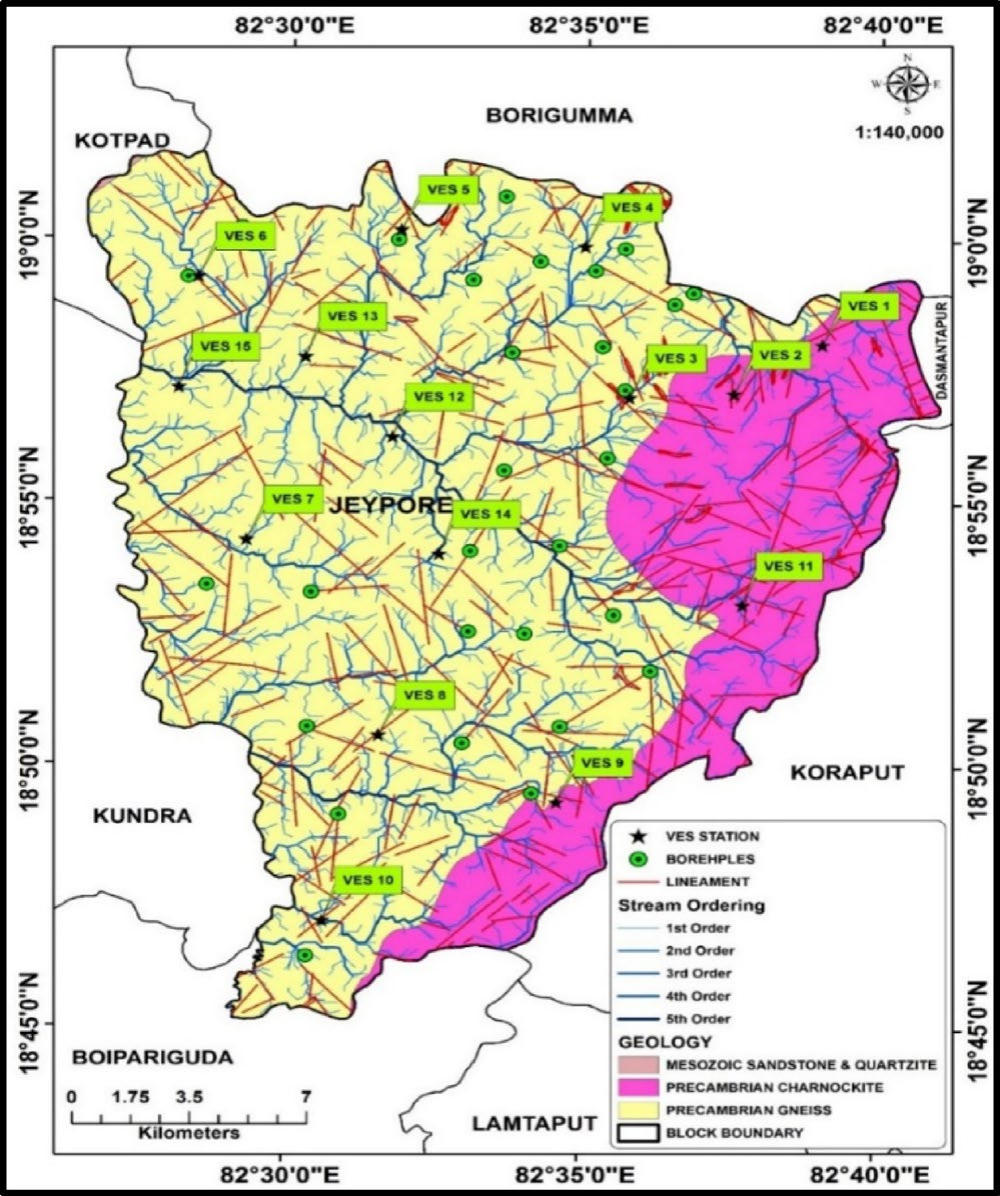
Geological framework, lineament distribution, stream ordering, and locations of VES stations and boreholes in the Jeypore region of Koraput district.

In this study, a wide array of spatial datasets has been systematically utilized to pinpoint and examine potential groundwater zones throughout the study area (S1). These datasets offer essential insights into the region’s hydrogeological, geomorphological, and structural features, facilitating a thorough spatial evaluation of groundwater availability (Fig. 2).

**Fig. 3:**
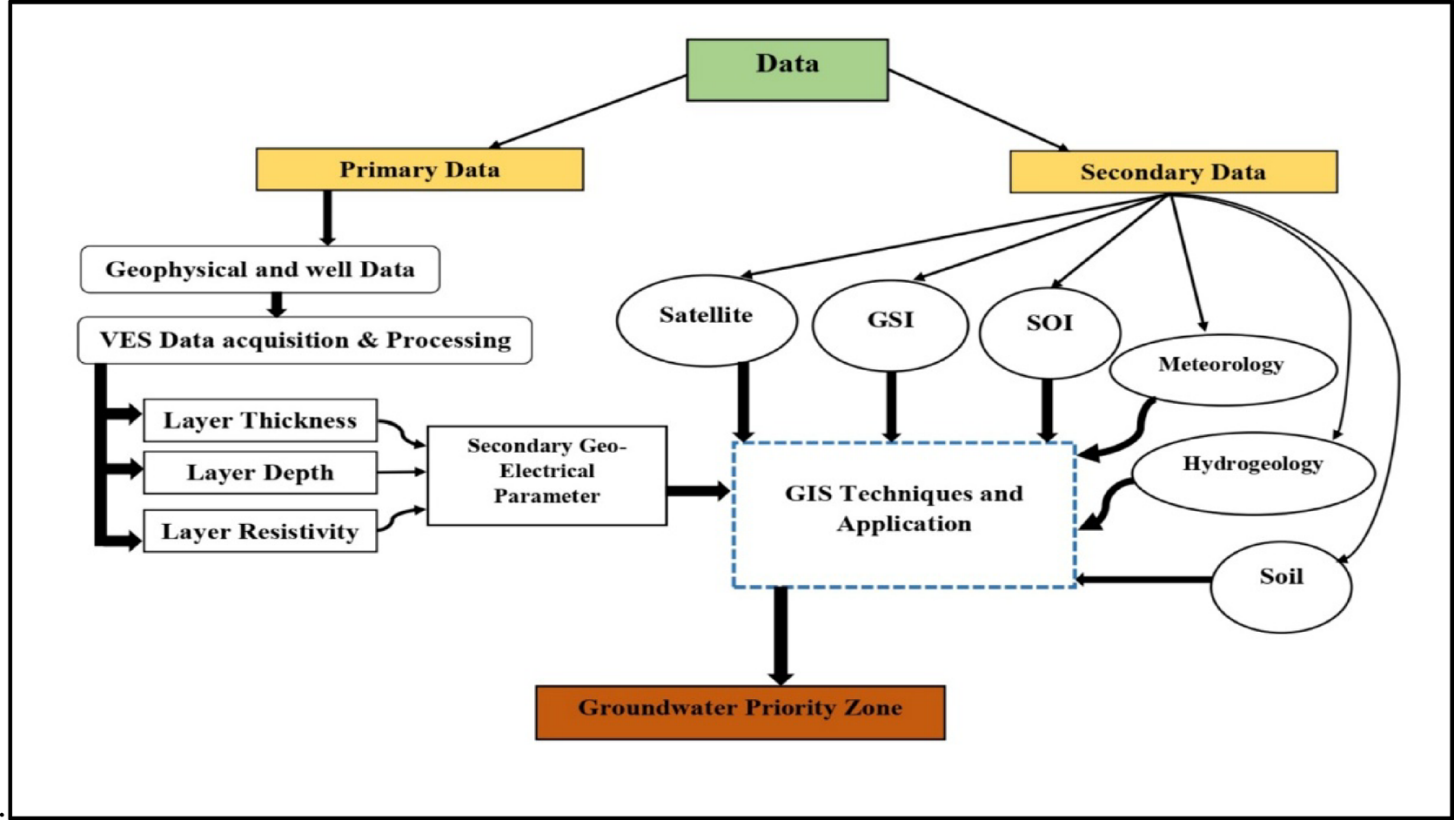
Conceptual framework illustrating the integration of VES-derived geoelectrical parameters with remote sensing and GIS-based secondary datasets (GSI, SOI, satellite, meteorology, hydrogeology, and soil) for delineating groundwater priority zones in the Jeypore region.

The study adopts an integrated approach, utilizing both primary and secondary data to explore the geological, hydrogeological, and environmental aspects of the area under investigation. Field-based primary data, including geophysical surveys (VES, resistivity profiling) and well inventories, were gathered to assess subsurface conditions and groundwater dynamics (Fig. 3). Supplementary secondary data from sources such as Landsat 8 Satellite images, SRTM images, GSI (Geological Survey of India), SOI (Survey of India), IMD (India Meteorological Department), and NBSS&LUP (National Bureau of Soil Survey and Land Use Planning), which include satellite imagery, geological maps, and meteorological records, contributed to a detailed spatial and temporal evaluation of groundwater potential.

### Geophysical and well inventory data based on VES techniques

Geophysical and well inventory data were collected using the Vertical Electrical Sounding (VES) technique to assess subsurface lithology, identify aquifer zones, and evaluate groundwater potential across the study area. Fifteen VES points were systematically distributed across the study area, with adjustments made for terrain features such as gullies and infrastructure. Data acquisition involved measuring voltage and current to calculate resistivity values that help delineate lithological boundaries and potential aquifers^48^.

The Vertical Electrical Sounding (VES) technique was employed to investigate subsurface lithology, delineate aquifer zones, and evaluate groundwater potential. Fifteen VES points were distributed across the study area, with minor adjustments made to avoid gullies and built-up structures. Field measurements were conducted using an ABEM Terrameter SAS1000 with the Schlumberger configuration, employing electrode spacings up to 800 m to investigate deeper subsurface layers. While we worked, we logged current and potential differences, ran the calculations to get apparent resistivity, and tracked how things changed beneath the surface^48,49^.

The acquired resistivity data were processed using the IPI2WIN software (version 3.0.1). The software uses smooth inversion, kind of like Occam’s approach: start with the simplest explanation, only add complexity if the data really calls for it. We began by assuming the ground was a simple, uniform layer with a resistivity of 100 Ω·m and a thickness of 10 m. Then, we set model constraints based on what we know about the area: topsoil thickness between 0.5 and 5 m (resistivities from 30 to 300 Ω·m). The inversion process was iterated up to 30 times for each dataset and terminated when the RMS error changed by less than 1% or when the χ² value approached unity or fractured zone going from 5 to 100 m deep (10 to 150 Ω·m); and bedrock underneath 50 m, above 150 Ω·m. We only kept models with an RMS misfit of 5% or less, and our results landed between 1.2% and 4.8%averaging at 2.9%. The interpreted resistivity and layer thickness values were subsequently used to calculate Dar–Zarrouk parameters. All VES data were interpreted using IPI2WIN (Version 3.0.1), which applies a smooth inversion approach conceptually like Occam’s inversion to obtain the simplest model consistent with the observed resistivity data. To ensure reproducibility, the manuscript now includes the initial model assumptions, layer constraints based on regional lithology, iterative optimization procedures, convergence criteria, and RMS misfit statistics. Furthermore, the low RMS error values (1.2–4.8%, average 2.9%) and the consistency between geoelectrical interpretations, pumping-test observations, and hydrogeological conditions demonstrate the robustness and reliability of the derived subsurface models.

With these solid inversion results, we pulled out resistivity and layer thickness values to calculate Dar-Zarrouk parameters like longitudinal conductance and transverse resistance. These numbers really help pin down aquifer characteristics and what’s happening below the surface, crucial details for solid groundwater assessment and smart management plans (Ibrahim et al.2023, Sarkar et al^50^).

In hydrogeophysical investigations, Dar–Zarrouk parameters derived from resistivity data are widely used to estimate the hydraulic characteristics of aquifers. Among these parameters, transverse resistance (T) plays a significant role because it has a strong correlation with aquifer transmissivity (T_r_) obtained from pumping tests. The transmissivity of an aquifer is calculated using the relation T_r_ = K × h, where *Tr* represents transmissivity (m²/day), *K* denotes hydraulic conductivity (m/day), and *h* is the aquifer thickness (m), highlighting the significant calculation used to evaluate groundwater flow capacity. Hydraulic conductivity can be further estimated from geoelectrical measurements using the empirical equation K = aρᵇ, where *ρ* is the aquifer resistivity and *a* and *b* are lithology-dependent constants determined from previous studies. Through these relationships, resistivity data obtained from Vertical Electrical Sounding (VES) can be quantitatively transformed into hydrogeological parameters, enabling a reliable assessment of aquifer potential in regions where direct pumping test data are limited.

Longitudinal conductance was assessed to evaluate the protective capacity of surface layers, particularly in clay-rich zones that inhibit pollutant infiltration (Niaz, et al., ^43^). Transverse resistance indicated the ability of permeable layers such as sand or gravel to transmit groundwater effectively. Average resistivity values were derived in both longitudinal and transverse directions to characterize anisotropic subsurface conditions, with lower longitudinal resistivity often indicating water-saturated or clayey zones. Finally, aquifer transmissivity, reflecting the aquifer’s capacity to transmit water, was estimated by combining hydraulic conductivity with aquifer thickness, providing key insights into groundwater yield potential critical for irrigation and supply.

The Inverse Distance Weighting (IDW) method was used in Surfer-25. The IDW method was chosen over other methods (e.g., kriging, spline, natural neighbor) for three reasons:

First, preliminary cross-validation using 20% of the VES data as test points demonstrated IDW (power parameter = 2) achieved the lowest root mean square error (RMSE = 12.4 Ωm for resistivity) compared to ordinary kriging (RMSE = 15.7 Ωm), spline (RMSE = 18.2 Ωm), and natural neighbor (RMSE = 14.9 Ωm).

Second, the limited number of VES points (*n* = 15) precludes robust variogram modeling necessary for kriging: geostatistical methods require > 30 sample points for reliable semi-variogram estimation (Wang, 2015). IDW is agnostic to the underlying spatial distribution of samples because it does not assume spatial stationarity or require variogram fitting. Therefore, IDW is better suited to sparse data.

The study area exhibits localized resistivity heterogeneity controlled by fracture systems and weathering patterns. Because the IDW is a distance-weighted local averaging method, IDW better preserves local extremes in resistivity than kriging might. In other words, kriging is likely to produce smoothed surfaces that under-sample small-scale aquifer features of interest for borehole targeting. The power parameter was tested at values of 1, 2, and 3; p = 2 gave the lowest cross-validation RMSE and did not produce excessively strong ‘bull’s-eye’ effects that result from higher power values. A variable search radius with 12 nearest neighbors was used to avoid unstable interpolation due to uneven point distribution.

The 1D resistivity inversion was carried out using the VES 3.0 inversion program, where the constraints for the layers were based on the observations made in the field and the knowledge of the lithology of the area, ensuring that the resistivity values for the different layers were realistic. The iterative modeling process ensured that the layer resistivity and thickness were adjusted in such a manner that the difference between the observed and calculated apparent resistivity curve values was minimized. This ensured that the model was adequately fitted despite the lack of information regarding the error percentage. The inversion process was carried out using the smooth modeling approach, which is similar to the Occam inversion method that seeks the simplest model that adequately fits the observed data. The Eastern Ghats hard-rock terrain exhibits complex geological conditions, where groundwater occurrence is strongly controlled by weathering and fracture systems. In this study, the Schlumberger VES method was adopted to investigate vertical aquifer characteristics and was supported by geological, geomorphological, hydrogeological, and pumping-test data to improve interpretation reliability. Although 2D ERT could provide additional information on lateral subsurface variations, the present integrated VES-based approach offers a practical and scientifically reliable framework for groundwater assessment across a large and topographically challenging region.

### Remote sensing and GIS techniques

To investigate spatial and temporal patterns in the study area, elevation data from the SRTM mission and DEMs were employed. A steady stream of satellite images, taken every 16 days as part of the Global System-2 framework, facilitated long-term monitoring and analysis. The combination of multispectral data from the Landsat 8 OLI and TIRS sensors, known for their advanced thermal and optical capabilities, significantly enhanced the identification of groundwater-related features. High-resolution datasets, including thermal bands, were integrated to improve the accuracy of environmental mapping. Utilizing ArcGIS 10.7, several thematic layers were created and overlaid to pinpoint areas with high groundwater potential. The selection of datasets was strategically aligned with research objectives and limited by data availability.

### Geospatial data acquisition and processing

Landsat 8 images were downloaded from the website “USGS Earth Explorer.” The images were utilized for the development of the land cover maps. ArcGIS 10.7 assisted in this regard. ArcGIS 10.7 was utilized for the development of topographical and drainage maps. The maps were developed using the Digital Elevation Model (DEM). The satellite images were subjected to radiometric and geometric corrections. Calibration and DOS were utilized for the improvement of image quality. The developed land cover maps have five different classes (Fig. iso-resistivity).

### Layering

The geology of the region has a major impact on the occurrence, movement, infiltration, and runoff of groundwater. The role of geomorphology was also verified through reference topographic maps in the groundwater recharge and distribution processes. The soil structure has a major impact on the infiltration capacity of the soil. Soils with fine particles have a low infiltration capacity, which results in low groundwater recharge potential^51,52^. Lineaments are long, narrow bands of geological features, usually the result of tectonic activity, which are critical in groundwater zones, as well as mineral and geothermal potential. The lineament density was computed through the GIS tool to understand the lineament density better. An area with high drainage density indicates low infiltration capacity^21,25^. Factors like topography, geomorphology, and land use have a positive correlation with drainage density. Rainfall data was collected through the Odisha rainfall monitoring system, which showed a range of 1562 mm to 1635 mm of rainfall in the area, which is important to assess the groundwater recharge potential. Slope has a major impact on the infiltration capacity of the soil. Steep slopes have high runoff potential, while flat surfaces have high infiltration potential.

The land use/cover was determined using satellite images, which revealed changes that might influence groundwater availability. The NDVI was used to measure vegetation density to infer groundwater potential. Dense vegetation is an indication of good groundwater conditions, as suggested by Chatterjee et al.^39^.

### Analytical Hierarchy Processes

The study incorporated thematic layers such as geology, geomorphology, soil, NDVI, lineament and drainage density, rainfall, slope, physiography, LULC, groundwater fluctuation, and hydrogeology, as these collectively influence groundwater occurrence, movement, storage, and recharge in the region. These layers encompass crucial local hydrogeological elements, including rock types, landforms, soil properties, vegetation cover, fracture networks, runoff and rainfall patterns, slope, and aquifer characteristics, which together dictate groundwater availability and distribution. Each parameter was evaluated against others using a standardized scale from equal to extremely important, ensuring a systematic judgment process^53^. The pairwise comparisons were normalized by dividing each element by the sum of its column, allowing for consistent weighting across all criteria (S2). Although the assignment of relative weights involved some subjectivity, it was guided by the hydrogeological characteristics of the Eastern Ghats Mobile Belt (EGMB). Groundwater occurrence in this hard-rock terrain is primarily controlled by secondary porosity associated with weathering and fracturing. Therefore, geology was assigned the highest weight (0.235) because lithology strongly influences weathering intensity, fracture development, and aquifer storage potential. Although other criteria are also very significant, they were assigned relative weight based on their secondary but significant roles in recharge, storage, and transmission (see pairwise matrix, S2). Eigenvector methods were then employed to calculate the principal eigenvalue and derive the final weights (S3), indicating the relative importance of each factor in assessing groundwater potential^25,54,55^. The Analytical Hierarchy Process (AHP) is a robust GIS-based tool frequently used to identify potential groundwater zones by evaluating multiple influencing factors^55^. In this study, twelve key parameters were chosen, and a pairwise comparison matrix was developed to assign relative weights (S4) to each criterion based on their significance^54^. To ensure the reliability of the weighting process (S5), the consistency ratio (CR) was calculated, indicating the degree of logical consistency in the pairwise comparisons. A CR value below 10% was obtained (S6), confirming acceptable consistency and validating the judgments used in the analysis^25,53^. The integration of all weighted thematic layers enabled a comprehensive evaluation of groundwater potential zones, supporting decision-making for resource management (Dandapat et al., 2024). This method provides an objective framework for combining diverse hydrogeological, climatic, and geomorphological data into a single priority map. The process helps identify zones with the highest likelihood of groundwater occurrence by systematically accounting for complex interactions among influencing factors (S7). The Analytical Hierarchy Process (AHP) was applied to evaluate twelve parameters: geology, geomorphology, soil, NDVI, lineament and drainage density, rainfall, slope, physiography, LULC, groundwater fluctuation, and hydrogeology, chosen for their influence on groundwater occurrence, movement, storage, and recharge. Expert judgment assigned relative weights through a pairwise comparison matrix using Saaty’s^53^standardized scale, which were then normalized and validated via principal eigenvalues and a consistency ratio below 10%. While the methodology follows Dar et al.^54^, additional factors such as NDVI, groundwater fluctuation, and slope were included to capture local hydrogeological, climatic, and terrain-specific variations. This approach provides a reliable and objective framework for identifying groundwater potential zones and supporting resource management decisions. Overall, AHP offers a robust and transparent approach for groundwater zoning, especially in regions where direct data is limited or difficult to obtain.

The weighting of thematic layers was based on the hydrogeological characteristics of the Eastern Ghats Mobile Belt, where geology exerts the primary control on groundwater occurrence through lithology, weathering, fracture development, and aquifer storage properties. The sensitivity analysis further confirmed this physical relationship, showing the highest influence for hydrogeology (4.8%), geology (4.1%), lineament density (3.5%), and groundwater fluctuation (3.2%), which are the key factors governing groundwater distribution in hard-rock terrains. These results demonstrate that the parameters assigned higher AHP weights have the greatest impact on model output, thereby validating the scientific basis of the weighting scheme. In contrast, the relatively low sensitivity of NDVI and LULC indicates that variations in these secondary parameters produce only minor changes in groundwater zonation, confirming the robustness and stability of the developed model.

### Groundwater potential index (GWPI)

The Groundwater Potential Index (GWPI) was calculated using the following equation (Eq. 1) (Zhran et al^55^ Chatterjee et al^39^).

1 $$\begin{aligned} GWPI{\text{ }} & = \{ \left( {GEO_{w} \times {\text{ }}GEO_{{wi}} } \right){\text{ }} + {\text{ }}\left( {GEOM_{w} \times {\text{ }}GEOM_{{wi}} } \right){\text{ }} + {\text{ }}\left( {SOI_{w} \times {\text{ }}SOI_{{wi}} } \right){\text{ }} + {\text{ }}\left( {NDVI_{w} \times {\text{ }}NDVI_{{wi}} } \right){\text{ }} \\ & + {\text{ }}\left( {LD_{w} \times {\text{ }}LD_{{wi}} } \right){\text{ }} + {\text{ }}\left( {DD_{w} \times {\text{ }}DD_{{wi}} } \right){\text{ }} + {\text{ }}\left( {RF_{w} \times {\text{ }}RF_{{wi}} } \right){\text{ }} + {\text{ }}\left( {SLP_{w} \times {\text{ }}SLP_{{wi}} } \right){\text{ }} \\ & + {\text{ }}\left( {PHY_{w} \times {\text{ }}PHY_{{wi}} } \right){\text{ }} + {\text{ }}\left( {LULC_{w} \times {\text{ }}LULC_{{wi}} } \right){\text{ }} + {\text{ }}\left( {GWF_{w} \times {\text{ }}GWF_{{wi}} } \right){\text{ }} + \left( {HYDG_{w} \times {\text{ }}HYDG_{{wi}} } \right) \\ \end{aligned}$$

where:$$GWPI = \sum i = 1n\left( {Wi \times wi} \right)$$

$\:n$ = total number of thematic layers (12).

$\:{W}_{i}$ = AHP-derived normalized weight of the *i*-th thematic layer (e.g., geology = 0.18, hydrogeology = 0.16).

$\:{w}_{i}$ = normalized weight assigned to the specific sub-class within the *i*-th layer (e.g., for geology: gneiss = 0.25, charnockite = 0.20).

The GWPI values were then classified into four groundwater potential zones (weak, moderate, strong, very strong) using the natural breaks (Jenks) optimization method in ArcGIS 10.7 to minimize intra-zone variance and maximize inter-zone differences(S8).

## Sensitivity analysis

Sensitivity Analysis (Map Removal Method): Sensitivity analysis on AHP weights by sequentially excluding each thematic layer and computing the GWPI again. Sensitivity (S) for layer *i* was calculated as:

$$S = \frac{{(GWPI_{{all}} GWPI_{{without\,i}} )/GWPI_{{all}} }}{{1/(n - 1)}} \times 100$$

where $\:GWP{I}_{all}$ is the index using all 12.

layers, $\:GWP{I}_{withouti}$ is the index after removing layer *i*,

and $\:n=12$. Results (Table S9) show that hydrogeology (S = 8.2%), geology (S = 7.6%), and lineament density (S = 6.9%) had the highest sensitivity, while NDVI (S = 1.2%) and LULC (S = 1.8%) had the lowest. This confirms that the model output is primarily controlled by physically relevant parameters rather than arbitrary weighting.

### Primary data based on the pumping

Pumping tests were performed on four wells to measure the drawdown (s) caused by a known pumping rate (Q), while observation wells recorded the aquifer response at different distances. The transmissivity (T) of the aquifer was calculated using the formula

$$\:T=\frac{Q}{s}({m}^{2}/hr)$$

which was then converted to m²/day by multiplying by 24. These measured transmissivity values were compared with theoretical estimates derived from Dar-Zarrouk parameters

$$\:{T}_{DZP}=(K\times\:b)$$

where K is the hydraulic conductivity and b is the aquifer thickness, to validate the VES-based aquifer characterization. The results showed that fractured granite zones generally have higher transmissivity than predicted, emphasizing the need for field verification through pumping tests (1). The estimation of hydraulic conductivity is now explicitly described using the empirical relationship K = aρᵇ, where ρ represents aquifer resistivity and the constants a and b were selected from published studies conducted in comparable hard-rock terrains. Transmissivity was subsequently calculated using the standard equation T_r_ = K × h, where h denotes the saturated aquifer thickness interpreted from the VES models, thereby establishing a quantitative linkage between geoelectrical and hydraulic parameters. To validate the results, pumping tests were conducted at selected VES locations and yielded transmissivity values ranging from 144 to 432 m²/day, while the Dar–Zarrouk-derived transmissivity values showed comparable spatial patterns across the study area. For example, at VES-14 the pumping-test transmissivity reached 432 m²/day, whereas the lowest value of 144 m²/day was recorded at VES-11, and the corresponding Dar–Zarrouk estimates showed reasonable agreement with these observations. The overall correspondence between VES-derived parameters, pumping-test results, groundwater potential zonation, and hydrogeological conditions supports the reliability of the adopted methodology for groundwater assessment in this complex hard-rock terrain.

### ROC-AUC model validation

The validation of the groundwater potential zonation model has been strengthened by incorporating the ROC curve (Fig. 8) and using 28 independent pumping-test wells, where yields > 5 L/s, 2–5 L/s, and < 2 L/s were classified as high, moderate, and low groundwater potential, respectively. The obtained AUC value of 0.86 with a 95% confidence interval of 0.78–0.94 indicates good predictive accuracy and strong agreement between model predictions and field observations. For spatial interpolation, IDW (power = 2) was selected after cross-validation because it produced the lowest RMSE (12.4 Ωm) compared with ordinary kriging (15.7 Ωm), natural neighbour (14.9 Ωm), and spline interpolation (18.2 Ωm). The combination of superior predictive performance, limited VES density (*n* = 15), and consistency with pumping-test results provides a robust justification for adopting the IDW approach and increases confidence in the generated spatial outputs.

## Results

The present study provides important insights into the structural and geodynamic characteristics of the study area based on surface topography and subsurface properties. The integration of terrain attributes with VES-derived subsurface characteristics improves understanding of aquifer systems and groundwater flow conditions. The variation of resistivity with the associated Dar-Zarrouk parameters (thickness, longitudinal conductance, and transverse resistance) can be a reliable method for estimating groundwater occurrence and guiding water resources management in the study area. The correlation between the results and the pumping test data confirmed the presence of a productive aquifer. The transmissivity value from the pumping test is greater than the transmissivity in fractured granite, which is calculated from Dar-Zarrouk parameters. The 3D models and iso-resistivity contour maps generated using Surfer-25 revealed spatial variations in layer thickness with depth. The thickness of the first layer exceeds 20.4 m in the west and thins towards the east to 0.6 m. The second and third layers are thickest in the south, attaining 73.6 m and 99.5 m, respectively, and thinning towards the west to 2.5 m and north to 14.2 m. The variation of resistivity has been represented on iso-resistivity maps that show the spatial heterogeneity of the resistivity. The resistivity values are up to 106.5 Ωm in the central part of the first layer and in the deeper layers, they are up to 695 Ωm. This signifies that the material in this region is compact or dry.

In hard rock hydrogeophysics, resistivity classifications are commonly used to identify aquifer and fractured zones. By considering the moderate values of resistivity, as they often indicate the presence of weathered and fractured zones. For example, resistivity values of 10 to 60 Ω·m often indicate the presence of weathered and water-saturated zones, whereas values of 60 to 150 Ω·m often indicate the presence of semi-weathered or fractured zones. Resistivity values significantly greater than 150 to 300 Ω·m often indicate the presence of hard rock or dry basement rock, which cannot be used as aquifer zones. These values have been identified by various recent VES studies for different terrains. Venkateswara et al. (2019b) reported resistivity values of < 10 Ω·m for the highly weathered/saturated zone, 10 to 60 Ω·m for weathered zones, 61 to 150 Ω·m for fractured zones, and values significantly greater than 150 Ω·m for hard rock. Similar resistivity ranges have been reported in previous geophysical studies conducted in hard-rock terrains. Resistivity values below 100 Ω·m generally indicate weathered and fractured zones with groundwater potential, whereas values greater than 100 Ω·m often indicate the presence of hard rock. Even though there are no previous geophysical studies conducted for the study area, the results of the present study are consistent with the experiences of the region, and the results of the present study can be used to classify the different VES layer values as aquifer and fractured zones or hard rock. If the resistivity values and their ranges are supported by the types of curves, then the results of the present study would become more reliable.

The key VES parameters were quantitatively integrated with lineament density and geomorphological mapping. Moderate resistivity zones (40–100 Ωm) with thick weathered/fractured layers (> 50 m) spatially coincide with areas of high lineament density (1.60–2.37 km/km²). For instance, VES point 4 and 14, which display HA-type curves and high transmissivity (432 m2/day), are within 250 m of the lineament intersection locations. In contrast, zones of very high resistivity (> 150 Ωm) with thinner weathered layers (< 15 m) correspond with the structural hills and zones of very low lineament density (< 0.83 km/km2), characteristic of compact, unweathered basement. Moreover, 78% of the “strong” and “very strong” groundwater potential zones are hosted by Pedi plain geomorphic units where the accumulation of weathering products on the gentle slopes (2.3–9.7°) has resulted in thick saprolite zones, observed by the VES surveys.

**Fig. 4:**
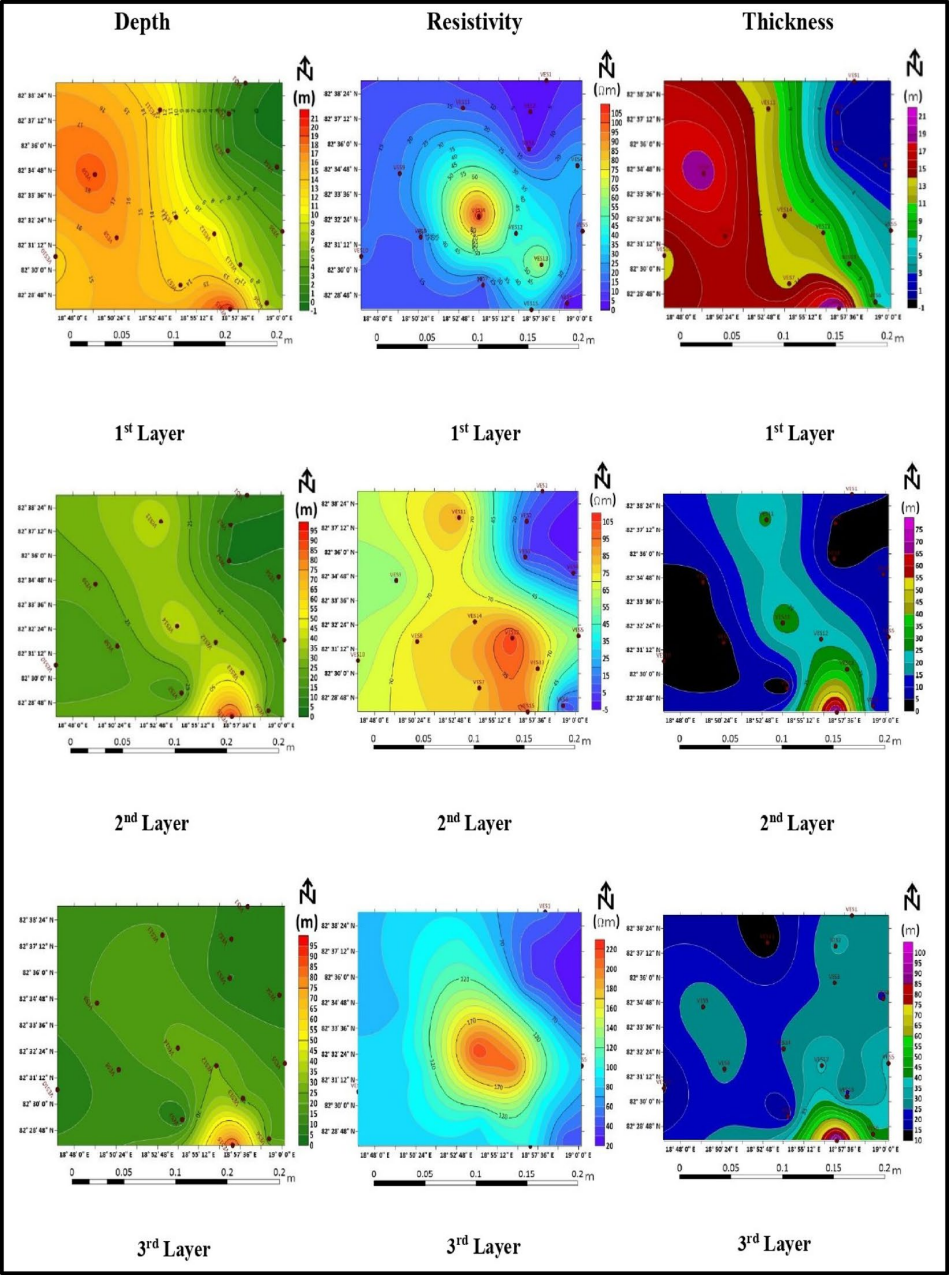
Spatial distribution maps of depth, resistivity, and thickness for the first, second, and third geoelectrical layers derived from VES data in the Jeypore region.

The subsurface layers exhibit significant spatial variation. The first layer is thickest in the western region (20.4 m) and gradually thins eastward to 0.6 m., while the second and third layers reach their maximum depths of 94 m and 193.5 m respectively in the southern region, indicating a substantial accumulation of geological materials (Fig. 4). These patterns suggest the southern part of the study area contains the most extensive and deeply developed subsurface formations, making it a zone of hydrogeological interest. The subsurface depth of ~ 193 m was interpreted using the Vertical Electrical Sounding (VES) method, which is specifically designed to investigate vertical variations in resistivity and layer thickness. The depth of investigation was achieved through sufficiently large electrode spacing, allowing reliable penetration to deeper formations (S10). The area is predominantly composed of laterally uniform sedimentary layers, where the sounding technique provides dependable results for identifying subsurface stratification. Electrical Resistivity Tomography (ERT) was not applied because the primary objective of the study was to determine vertical layering rather than detailed lateral variations. Therefore, the VES sounding approach was considered more appropriate, efficient, and adequate for achieving the hydrogeological objectives of the investigation.

RockWorks-17 is a valuable software tool for analyzing lithology, stratigraphy, aquifers, and creating lithological bar graphs. It is particularly well-suited for processing data from Vertical Electrical Sounding (VES) surveys (S9). The software enables detailed visualization and interpretation of subsurface geological formations. Its versatile features make it an essential resource for geologists and hydrogeologists working on groundwater and stratigraphic studies.

The analysis of Vertical Electrical Sounding (VES) suggests that in the Eastern Ghat’s hard rock region, groundwater is predominantly found in the weathered laterite and fractured granite layers, with minimal primary porosity in the bedrock. Optimal drilling sites are mainly located in valleys and gently sloping terrains where weathered and fractured granite layers exceed 30–60 m in thickness, as these areas offer greater storage capacity and transmissivity. Borewell depths are anticipated to range from 80 to 120 m in moderate zones and can reach 150–200 m in high-potential areas such as Solopa and Konga, with yields fluctuating between 0.5 and 6 L/s based on aquifer thickness and fracture density. Transmissivity (T), a crucial hydrogeological parameter, is calculated as T = K × b, where K represents hydraulic conductivity and b is the saturated thickness; for instance, with K = 1.5 m/day and b = 40 m, T equals 1.5 × 40 = 60 m²/day, indicating a moderate aquifer potential suitable for sustainable groundwater development.

The inversion gave steady results at every VES station. RMS misfit values fell between 1.2% at VES-04 and 4.8% at VES-11. On average, the RMS misfit landed at 2.9%, which shows the modeled resistivity curves line up well with what was seen in the field. This supports the reliability of the subsurface layering that was interpreted.

**Fig. 5:**
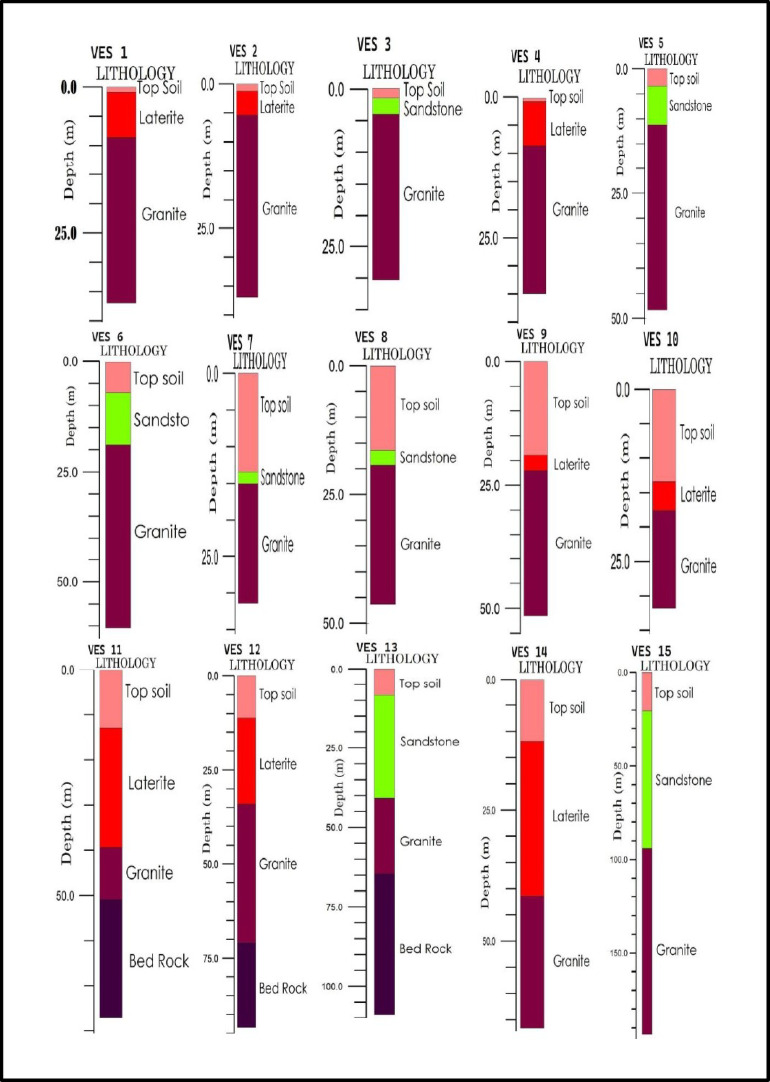
Combined lithological column and VES depth-resistivity data showing subsurface stratification across representative locations in the study area.

Fifteen VES sites were randomly chosen throughout the study area to assess subsurface lithology variations, uncovering distinct sequences of topsoil, laterite, sandstone, granite, and occasionally super hard bedrock. For instance, VES-01 near Singhibandha and VES-02 at Kebidi are predominantly composed of granite, making up over 79% and 84% of their respective profiles, whereas VES-07 and VES-08 exhibit thicker topsoil and sandstone-laterite layers, suggesting shallower weathering profiles. The deepest subsurface formations were identified at VES-11, VES-12, and VES-13, where super-hard bedrock was found beneath extensive sequences of granite and lateritic materials, reaching depths of up to 108.9 m. These results are visually represented through lithology bar graphs created using RockWorks-17 software, providing a detailed depiction of the vertical stratigraphy across the region (Fig. 5).

IPI2WIN software was specifically used for plotting and processing geo-electrical data obtained from Vertical Electrical Sounding (VES) surveys. Labelled diagrams showing typical three- to four-layered sounding curves were created for VES points 1 through 15. The software facilitated the interpretation of true resistivity and the corresponding thicknesses of various subsurface layers. These extracted geo-electrical parameters provided a clear understanding of the subsurface structure and aided in identifying different geological formations (S9).

Vertical Electrical Sounding (VES) points 1, 2, 3, 5, 6, 7, 8, 9, 10, 11, 12, 13, and 15 exhibit a resistivity pattern of ρ₁ < ρ₂ < ρ₃, which corresponds to the AAA curve type (S9). This curve indicates a continuous increase in resistivity with depth, often interpreted as a transition from conductive surface materials to highly resistive basement rock. The first layer typically consists of topsoil or weathered materials with low resistivity resulting from moisture or clay content, while the second layer comprises moderately resistive laterite, semi-weathered rock, or dry sand. The third and deepest layer generally exhibits high resistivity, indicating compact and dry crystalline bedrock like granite or gneiss, which limits deep aquifer development and confines groundwater availability to the overlying weathered zone.

**Fig. 6:**
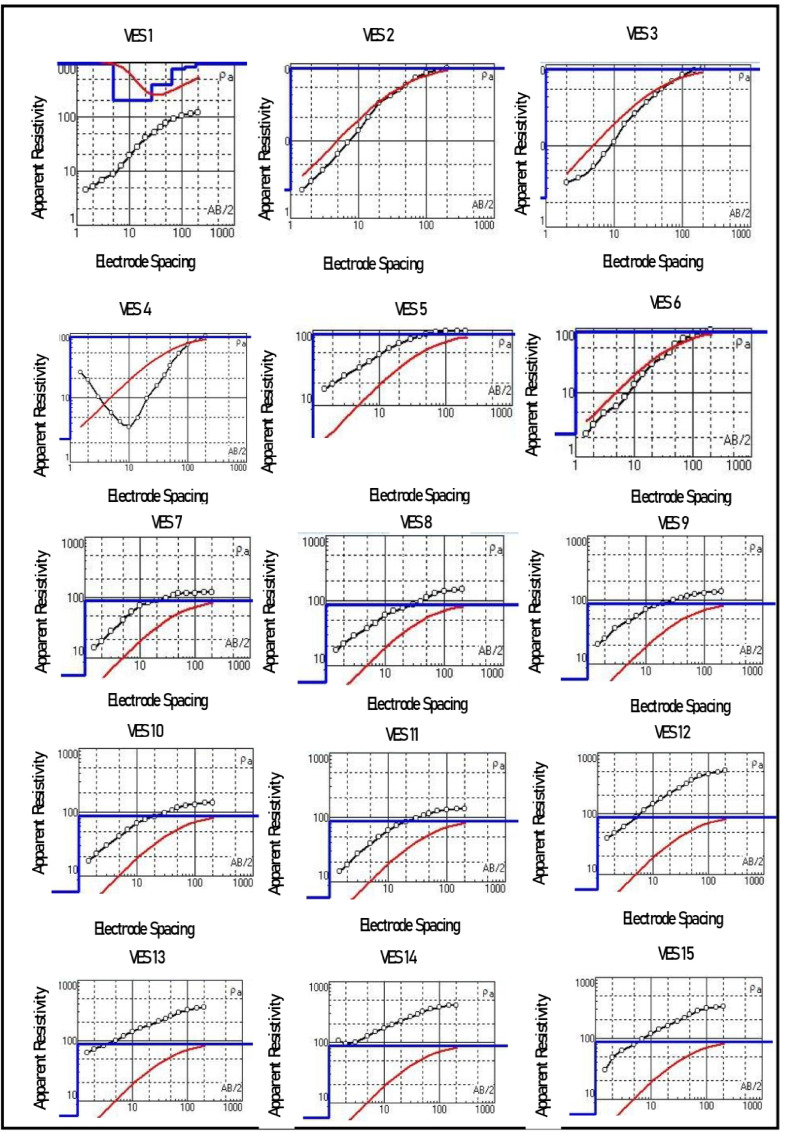
Peak voltage positions (V) recorded for Vertical Electrical Sounding (VES) stations 1–15 across the study area, showing consistent background values (100 V) with variable peak positions (102–118 V) indicating subsurface electrical heterogeneity and potential zones of contrasting lithology or groundwater saturation.

In contrast, VES points 4 and 14 follow a ρ₁ > ρ₂ < ρ₃ < ρ₄ resistivity pattern, corresponding to the HA curve type. The HA curve type begins with a resistive surface layer, followed by a conductive second layer, often indicative of a water-saturated zone like clay or weathered material, and then transitions into more resistive third and fourth layers (S9). This configuration is characteristic of multi-layered groundwater systems, where both shallow and deep aquifers may coexist within weathered and fractured formations. Compared to the AAA curve, the HA type generally indicates better groundwater potential, especially when the middle conductive layer is thick and saturated (Fig. 6).To improve the reliability of the interpretation, the inversion results obtained from the IPI2WIN software were evaluated by examining the fitting error between the observed and calculated resistivity curves for each VES model. The root mean square (RMS) misfit of the inversion for the fifteen VES locations remained within an acceptable range, generally below about 5%, indicating a good agreement between the field data and the modeled responses. This low inversion error confirms that the estimated resistivity values and layer thicknesses provide a realistic representation of the subsurface conditions. Minor uncertainties may arise due to lateral heterogeneity of geological formations and field measurement limitations, which are inherent to resistivity sounding methods. Nevertheless, the consistent curve fitting and low RMS errors across all VES points support the reliability of the interpreted lithological sequences and depth estimations.

The interpretation of resistivity data was carried out by correlating field-derived apparent resistivity values with established resistivity ranges for typical lithological units of hard rock terrains. Based on previous studies in similar geological settings and local hydrogeological conditions, different subsurface layers were delineated using characteristic resistivity ranges. In addition, curve types obtained from VES sounding were classified following standard resistivity curve nomenclature, where variations in layer resistivity (ρ₁, ρ₂, ρ₃, …) determine curve geometry. In this study, AAA-type curves represent a progressive increase in resistivity with depth (ρ₁ < ρ₂ < ρ₃), indicating a transition from weathered to compact formations, whereas HA-type curves (ρ₁ > ρ₂ < ρ₃) reflect the presence of an intermediate low-resistivity layer, typically associated with saturated/weathered or fractured zones overlying a resistive basement. These classifications aid in identifying aquifer horizons and subsurface heterogeneity (Fig. 6).

Analyzing resistivity data to identify groundwater priority zones (GWPZ) was greatly enhanced by the use of computer software, with ArcGIS 10.7 playing a vital role in spatial analysis. Incorporating 12 subsurface layers ensured a thorough evaluation of the hydrogeological framework, allowing for accurate mapping and a better understanding of groundwater potential across the study area.

**Fig. 7:**
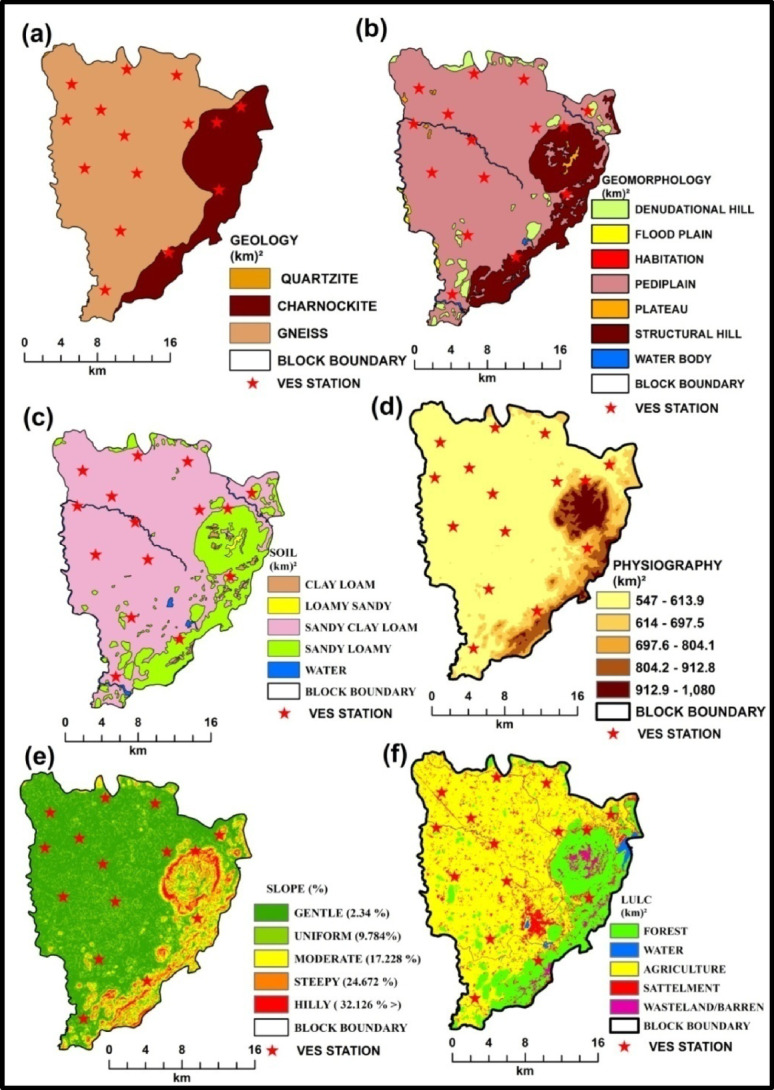

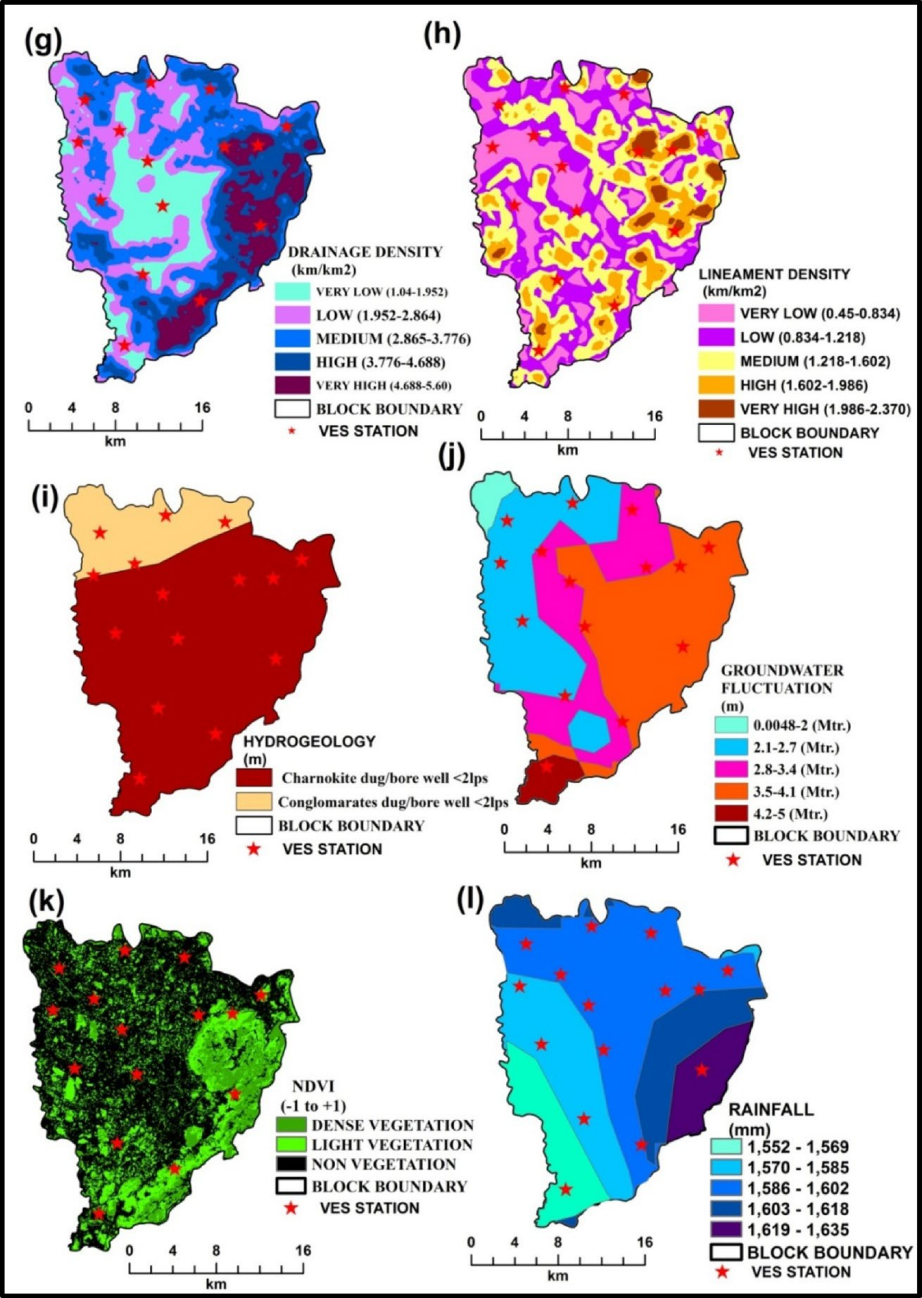
Thematic layers used for groundwater potential zone delineation in Jeypore Block: (**a**) Geology, (**b**) Geomorphology, (**c**) Soil, (**d**) Physiography, (**e**) Slope (%), (**f**) Land Use/Land Cover (LULC), (**g**) Drainage Density, (**h**) Lineament Density, (**i**) Hydrogeology, (**j**) Groundwater Fluctuation, (**k**) Normalized Difference Vegetation Index (NDVI), and (**l**) Rainfall distribution. Maps show the spatial distribution of each parameter with class-wise areal extent (km²), block boundary, and locations of Vertical Electrical Sounding (VES) stations.

The dominant geological formation in the study area is gneiss, covering approximately 344.11 square kilometres, which accounts for 75.46% of the total area. In contrast, charnockite is primarily found in the eastern part, occupying about 111.38 square kilometres, representing 24.43% of the region (Fig. 7a). Geology has been assigned the highest weight because it fundamentally controls the aquifer characteristics, including rock type, fracture density, and permeability, which directly influence groundwater occurrence and movement. Other criteria, such as geomorphology, soil, and slope, are important, but their influence on groundwater potential is secondary to the underlying geological framework. The interpretation of resistivity data has been strengthened through its integration with geological structures, lineament density, and geomorphological characteristics of the Eastern Ghats Mobile Belt. The results show that moderate resistivity values of 40–100 Ωm, representing weathered and fractured aquifer zones, are closely associated with areas of high lineament density ranging from 1.60 to 2.37 km/km². In contrast, highly resistive zones exceeding 150 Ωm correspond to compact basement rocks and areas of low lineament density, indicating poor groundwater potential. Furthermore, the most productive groundwater zones are concentrated within pediplains and valley-fill units, where gentle slopes and prolonged weathering have produced thick saprolite and fractured horizons that enhance groundwater storage and recharge.

Geomorphologically, the area exhibits a complex landscape, with pediplains being the dominant feature, covering approximately 341.24 square kilometres or 74.83% of the total area, distributed randomly throughout the region except in the east. Structural hills are mainly concentrated in the eastern part, occupying around 84.71 square kilometres, which accounts for 18.58% of the study area (Fig. 7b).

The study area features four soil types, with sandy clay loam being the most widespread, covering 348.4 square kilometres or 76.40% of the region, mainly outside the eastern part. Sandy loam soil dominates the eastern area, accounting for 22.50% of the total, which is approximately 102.3 square kilometres (Fig. 7c).

The physiography of the study area is divided into five distinct landforms. Valley undulating plains, ranging from 547 to 613 m in elevation, dominate the region with 334.2 sq.km (73.29%), followed by pediplains (614–697.5 m) covering 46.11 sq.km (10.11%), plateaus (697.6–804.1 m) at 27.04 sq.km (5.93%), undulating plateaus (804.2–912.8 m) at 30.12 sq.km (6.61%), and mountain chains (912.9–1080 m) occupying 18.53 sq.km or 4.06% of the area (Fig. 7d).

The slope of the study area is categorized into five types, with gentle slopes ranging from 2.34 to 9.784 degrees covering the majority of the region—approximately 282.78 sq.km or 62.01%. In contrast, steep slope hill areas, ranging from 32.116 to 39.56 degrees, occupy a much smaller portion, totalling 8.59 sq.km or 1.88% of the area (Fig. 7e).

The Land Use and Land Cover (LULC) classification of the study area includes five major categories. Agricultural land dominates, covering 291.7 sq.km or 63.97% of the total area, followed by forest land at 131.8 sq.km (28.90%). Settlements occupy 24.5 sq.km (5.37%), while waterbodies account for 6 sq.km (1.32%), and waste land or barren areas cover the smallest portion, with just 2 sq.km (0.44%) (Fig. 7f).

The drainage density of the study area is classified into five categories: very low, low, medium, high, and very high. Very low density (1.04–1.952) covers 56.37 sq.km (12.36%), low density (1.952–2.864) spans 122.13 sq.km (26.78%), medium density (2.864–3.776) accounts for 55.18 sq.km (12.10%), high density (3.776–4.688) covers 121.11 sq.km (26.56%), and very high density (4.688–5.60) occupies 101.21 sq.km, representing 22.20% of the total area (Fig. 7g).

The lineament density of the study area is categorized into five classes: very low, low, medium, high, and very high. Very low density (0.45–0.834) covers 95.15 sq.km (20.87%), low density (0.834–1.218) spans 142.25 sq.km (31.20%), medium density (1.218–1.602) accounts for 71.32 sq.km (15.64%), high density (1.602–1.986) covers 131.26 sq.km (28.79%), and very high density (1.986–2.37) occupies 16.02 sq.km, representing 3.51% of the total area (Fig. 7h).

The hydrogeology of the study area is primarily composed of two major rock types. Precambrian charnockite dominates the region, covering approximately 375.21 sq.km or 82.28% of the total area, while Precambrian conglomerates occupy 80.79 sq.km, accounting for 17.72% of the region (Fig. 7i).

Groundwater fluctuation in the study area is categorized into five ranges based on depth variations. The largest area falls within the 3.5–4.1 m range, covering 178.60 sq.km (39.17%), followed by 2.10–2.70 m at 141.71 sq.km (31.08%), 2.8–3.4 m at 107.28 sq.km (23.53%), 4.2–5.0 m at 17.21 sq.km (3.77%), and the least fluctuation, between 0.0048 and 2.00 m, occupying only 11.20 sq.km (2.46%) (Fig. 7j).

The NDVI classification of the study area reveals three distinct vegetation types. Non-forest land covers the majority, with 319.10 square kilometres (69.98%), followed by light forest at 107.18 square kilometres (23.50%), and dense forest occupying 29.72 square kilometres, which accounts for 6.52% of the area (Fig. 7k).

Rainfall in the study area is classified into five categories, with the highest category—excessive rainfall—covering approximately 105 sq.km, which accounts for 23.03% of the total area and is primarily located in the eastern region. Moving toward the southwestern part of the study area, rainfall intensity gradually decreases, indicating a spatial variation in precipitation patterns (Fig. 7l).

### Analytical Hierarchy Process (AHP)

The Analytical Hierarchy Process (AHP) facilitated the identification of groundwater potential zones by assigning and normalizing weights to twelve key parameters, with a consistency ratio of 2.36% confirming the reliability of the weighting scheme used in the multi-criteria evaluation.

### Groundwater priority zones

**Fig. 8:**
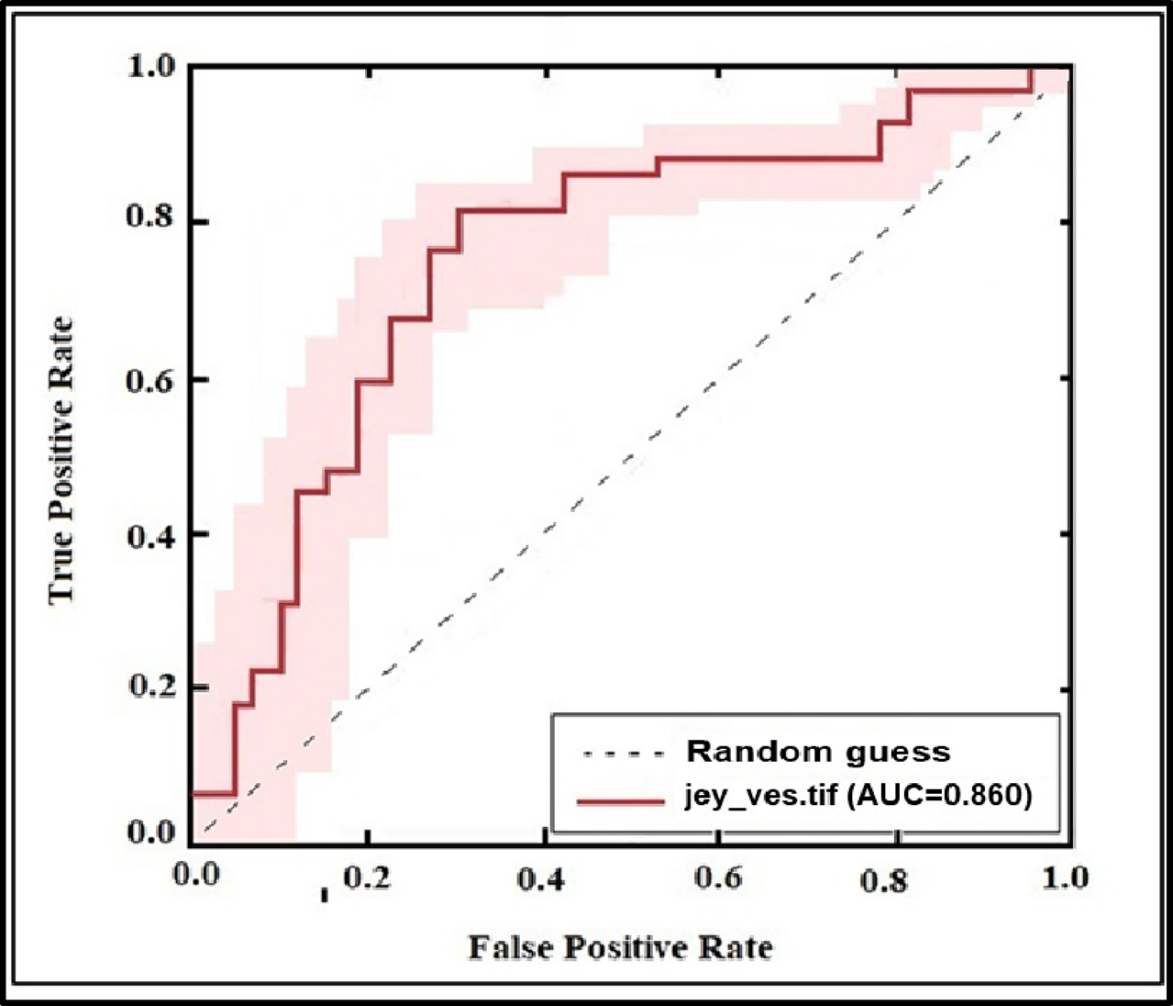
Receiver operating characteristic (ROC) curve validating the groundwater potential zonation model based on 28 independent pumping test wells (AUC = 0.86, 95% CI: 0.78–0.94). The diagonal dashed line represents random prediction (AUC = 0.5).

The delineation of Groundwater Potential Zones (GWPZ) in the study area was effectively achieved through the integration of geo-electrical data from Vertical Electrical Sounding (VES) and advanced geophysical software like Surfer-25, RockWorks-17, and IPI2WIN. These tools facilitated the generation of iso-resistivity maps and 3D subsurface models, revealing significant resistivity variations that distinguished between aquifer-bearing zones and hard basement formations. The Dar-Zarrouk parameters derived from VES datasuch as longitudinal conductance and transverse resistanceproved essential in evaluating groundwater storage and transmission potential. Areas with HA-type curves, marked by a conductive middle layer, were identified as favorable for groundwater occurrence, primarily concentrated in the eastern part of the study area. The integration of VES interpretations with AHP-weighted thematic layers in ArcGIS ensured a comprehensive and hydrogeologically sound mapping of groundwater potential zones (Fig. 8).

### Validation

The reliability of the groundwater potential zonation model was evaluated using the Receiver Operating Characteristic (ROC) curve and the Area Under the Curve (AUC) method. The ROC analysis compares the predicted groundwater potential zones with observed well-yield data, where wells yielding > 5 L/s were classified as ‘high potential,’ 2–5 L/s as ‘moderate potential,’ and < 2 L/s as ‘low potential.’ These classification criteria were established based on regional hydrogeological standards for hard-rock aquifers (CGWB, 2024).

The obtained AUC value of 0.86 (95% confidence interval: 0.78–0.94) indicates strong agreement between the model results and field observations, reflecting a high prediction capability. Generally, AUC values > 0.9 represent excellent accuracy, 0.8–0.9 good accuracy, 0.7–0.8 fair accuracy, and < 0.7 poor accuracy.

### Sensitivity analysis of GWPZ analysis

The results indicate that the GWPZ model is most sensitive to the removal of the hydrogeology (HYDG, mean sensitivity = 4.8%) and geology (GEO, mean sensitivity = 4.1%) layers, followed by lineament density (LD, 3.5%) and groundwater fluctuation (GWF, 3.2%). This has two implications. First, it confirms the logical consistency of the AHP weight assignment, as layers with the highest assigned weights (geology, hydrogeology) respond with the greatest influence on model output, thus confirming that the layers’ physicochemical characteristics was aligned correctly with the expert’s judgement. Second, the high sensitivity to LD and GWF indicates that structural discontinuities and dynamic recharge processes are crucial in this hard-rock terrain, which may not be obvious from the purely qualitative interpretation. In contrast, the model is little sensitive to layers such as LULC and NDVI (mean sensitivity < 1.5%) (S9).

Pumping tests carried out on the selected VES locations provided vital information on the hydraulic properties of the underlying fractured granite aquifers. The detailed results are presented in (S11). The discharge rates (Q) varied from 3 m³/hr at the Mohulobhota location (VES 11) to 9 m³/hr at the Ekomba location (VES 14). The drawdown in the observation wells varied from 0.12 m to 0.25 m, with corresponding recovery measurements. The lithological logs confirmed the presence of the fractured P3 layer in all the locations, with the VES 14 location categorized as highly fractured.

Subsequent values of Transmissivity (T) were obtained from the pumping tests carried out on the selected VES locations, as indicated in (S12). With a consistent drawdown (s) of 0.5 m used in the calculation, the obtained T values indicated a positive correlation with the discharge values. The T values varied from 6 m²/hr (144 m²/day) at the VES 11 location to 18 m²/hr (432 m²/day) at the highly productive VES 14 location.

A comparative analysis of the Transmissivity values obtained from the Dar Zarrouk parameter (DZP) and from pumping tests is provided in (S13). The table shows that the Transmissivity values obtained from the pumping tests are generally higher, with notable differences in the values obtained from the VES 1, 4, and 14 locations. The differences are attributed to the presence of fractures, which increase the bulk hydraulic conductivity of the aquifer, a phenomenon not fully captured in the DZP method. The Transmissivity values obtained from the DZP method were closest to the values obtained from the pumping tests at the VES 11 location, with the pumping test T value of 144 m²/day roughly matching the DZP T value of 101.8 m²/day.

**Fig. 9:**
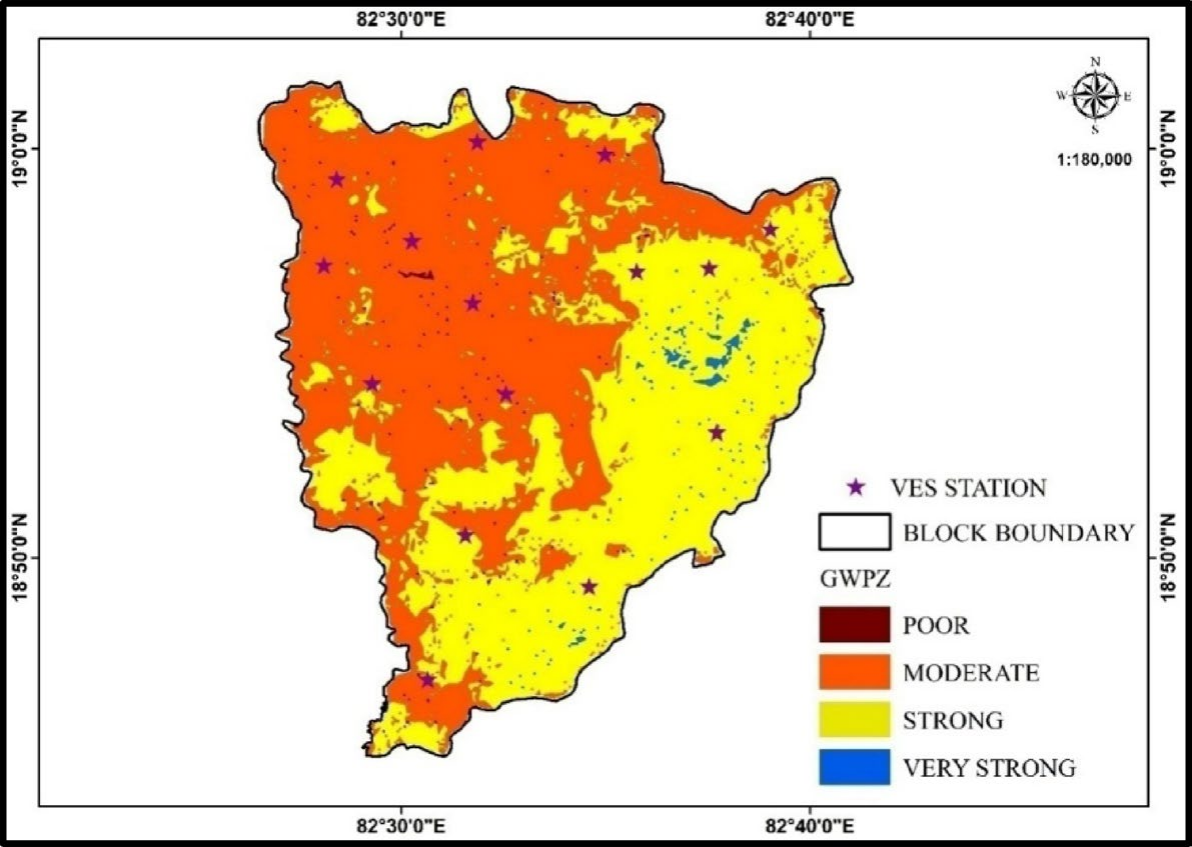
Groundwater Potential Zone (GWPZ) map showing spatial distribution of poor, moderate, strong, and very strong groundwater potential areas with locations of Vertical Electrical Sounding (VES) stations within the study block boundary.

The lithological interpretation of subsurface layers derived from VES data was carried out using the resistivity ranges presented in S14. The identified curve types (AAA and HA) further supported the delineation of weathered, fractured, and compact zones (Fig. 9).

## Discussion

The VES interpretation indicates that groundwater occurrence in the Jeypore Block is primarily controlled by the thickness and resistivity characteristics of weathered and fractured crystalline rocks common in the Eastern Ghats hard rock terrain, which largely influence groundwater occurrence in the Jeypore Block. The resistivity values obtained in this study indicate that there are three main layers of hydro stratigraphy in this area: a thin layer of topsoil–lateritic cover, a middle-weathered zone, and a third layer of fractured granitic rocks. Moderate resistivity values (40–100 Ωm) in the third layer indicate the presence of fractured granite aquifers with significant secondary porosity. Such hydrogeological characteristics are common in hard rock terrain in Odisha and other parts of peninsular India, where groundwater occurrence is largely confined to weathered mantles^25,47^; Chatterjee et al^39^). Similar geophysical studies conducted in hard rock terrain in other parts of eastern India also indicate that resistivity values ranging from 10 to 60 Ωm are indicative of highly weathered or saturated zones in this region, whereas those ranging from 60 to 150 Ωm indicate zones of fractured or semi-weathered zones of high groundwater potential^15,23^.

### Structural and geomorphological controls on resistivity signatures

The integrated interpretation of VES data with geomorphological and lineament analyses provides important insights into aquifer development. VES data were integrated with lineament and geomorphological analyses to better understand aquifer development within the Jeypore Block. Moderate resistivity values (40–100 Ωm) in the third layer indicate fractured granite aquifers containing groundwater within secondary porosity zones (1.60–2.37 km/km^2), suggesting that tectonic discontinuities have provided a preferential pathway for meteoric water percolation, leading to chemical weathering along the fracture planes. This is manifested in the thick (30–60 m) weathered-fractured zone observed in the southern and eastern parts of our study area. On the other hand, the zones of very low lineament density show massive unfractured bedrock. The latter is associated with uniformly high resistivity (> 300 Ωm) even at shallow depths. We also find that the geomorphic setting is equally significant. Pediplains and valley fills are associated with low topographic gradient, and thus there is more time for water residence, allowing for deeper weathering profiles, as manifested in the thick intermediate layers in the VES models associated with HA type curves. On the other hand, geomorphic hills (steep slope, > 15°) promote fast runoff, limiting weathering and as manifested in the thin AAA type resistivity profiles, the resistive basement is reached at shallow depth. The results suggest that the most productive aquifer zones occur where major lineaments intersect pediplain and valley-fill geomorphic units.

These structural features are helpful in facilitating infiltration and groundwater movement by increasing permeability in the fracture zones. This has been identified in several basement complexes in India, where groundwater potential is strongly related to fracture zone connectivity and geomorphic depressions^21,22,51^. In the Jeypore landscape, pediplains, valley fill, and gently sloping terrain are identified as relatively low resistivity and high layer thickness, indicating favorable conditions for groundwater recharge. Structural hills and steeper slopes are identified as high resistivity values, indicating compact rock layers and poor groundwater storage potential. These observations are consistent with regional hydrogeological studies in the Odisha region, where it has been identified that pediplains and valley fill units in the geomorphic landscape are typically favorable for groundwater development due to their greater weathered mantle and infiltration potential^47^.

The hydrogeophysical results provide important insights for groundwater development in the Jeypore Block. The zones of high productivity in drilling are likely to be located in the areas where the combined thickness of weathered and fractured zones exceeds 30–60 m and where intersections of lineaments occur over low-slope geomorphic surfaces such as pediplains and valley fills. Borewells drilled in these zones are likely to encounter fractured granite aquifers between 25 m and 70 m depths, as inferred in the VES interpretation, where the most productive resistivity layers are present. The pumping test validation also confirms the hydrogeophysical interpretation, where the transmissivity values vary between 144 and 432 m²/day, and the highest values are present in the highly fractured granite zones. These transmissivity values are consistent with moderate to high groundwater productivity, typical of fractured crystalline rock aquifers in peninsular India^33,50^. The hydrogeophysical interpretation and pumping test data, therefore, suggest that the borewell yields in favorable zones are likely to vary between 3 and 9 m³/h, depending on the intensity and thickness of the fractured zones in the hard rock terrain of southern Odisha^39,47^. The resistivity structures identified in the VES interpretation, therefore, offer a reliable approach for identifying the best locations for drilling, improving the success rates in groundwater exploration, and developing groundwater resources in the hard rock terrain of southern Odisha.

### Comparative analysis with Indian hard-rock terrain studies

The key hydrogeophysical parameters summarized in Table 5 are compared with those of other hard-rock terrains in India. While the weathered granite (40–100 Ωm) and compact bedrock (> 150 Ωm) resistivity ranges match broadly with the counterparts from the Aravalli^15^, and Deccan Traps^33^, the transmissivities (144–432 m^2^ day-1) in the Jeypore Block are moderate to high compared to the khondalitic terrains in the EG (e.g., 50–200 m^2^ day-1^47^. This is likely attributable to a dual-porosity system (intergranular in weathered saprolite and fracture-dominated in the underlying bedrock) that our VES interpretive framework (notably HA-curves) can capture. Notably, our VES points in the Jeypore Block show a lower proportion of AAA curves (vs. studies in the relatively homogeneous granite-gneiss of Karnataka^50^, but a significant number of HA-type curves (13% of VES points), reflecting a vertically more stratified hydrogeological system. This is expected given the poly-deformed EGMB lithologies (e.g., alternating bands of charnockite and khondalite) that lead to anisotropic weathering patterns. Our integrated resistivity-AHP approach and the field validation of the resultant maps, therefore, provide a transferable framework that is especially useful in complex, heterogeneous hard-rock terrains where resistivity interpretation alone is likely to be insufficient or misleading in determining the underlying hydrogeology.

Comparing the present results with recent hydrogeophysical studies conducted in hard-rock terrains of Odisha^39^, Karnataka^56^, the Aravalli region^57^, and the Deccan Traps (Kale et al.^58^) during the last five years. The moderate resistivity range of 40–100 Ωm identified in the Jeypore Block corresponds well with weathered and fractured aquifer zones reported from similar crystalline-rock environments. In contrast, resistivity values exceeding 150 Ωm indicate compact basement formations with limited groundwater potential^59^. The transmissivity values obtained from pumping tests (144–432 m²/day) are comparable to those reported from productive fractured aquifers in other parts of peninsular India, confirming the significance of secondary porosity and fracture connectivity in groundwater occurrence. However, the Eastern Ghats Mobile Belt exhibits additional geological complexity due to its poly-deformed khondalite–charnockite–gneiss assemblages, which create highly heterogeneous weathering and fracture patterns. Therefore, the integrated VES–GIS–AHP framework developed in this study provides a reliable approach for groundwater exploration and sustainable resource management in structurally complex hard-rock terrains across India^60^.

The groundwater potential zones are divided into five classes, with very strong and strong zones mainly found in the eastern region. Similar research is located on the Shatt Al-Arab Basin (Allafta et al.^4^,). Poor zones are scattered across various parts of the study area. Geological features like fractured and weathered charnockite in hilly terrain enhance groundwater recharge; similar research is situated on the Korba Coalfield, Central India (Singh et al.,2018). Surfer-25, RockWorks-17, and IPI2WIN software together enabled accurate subsurface modeling and VES data interpretation^23^.

Groundwater Potential Zones (GWPZ) were identified using geo-electrical data from Vertical Electrical Sounding (VES) and analyzed with Surfer-25, RockWorks-17, and IPI2WIN software, similar to research on the West Coast of India^61^. These tools helped model subsurface resistivity and lithology, highlighting aquifer formations where HA-type curves showed water-saturated layers, as seen in South-west Nigeria^62^. VES results matched spatial analysis in ArcGIS, revealing that fractured and weathered charnockite areas aligned with strong GWPZ in the east, similar to findings in the Kadugli district, Sudan^63^. Combining geophysical data with GIS ensured accurate groundwater mapping, important for sustainable water planning, as supported by research in Narmada District, India^64^, Kotagarh, Kandhmal, Odisha (Chatterjee et al.^51^).

The present study advances existing hydrogeophysical approaches by demonstrating the effective integration of Dar–Zarrouk parameters with GIS-based multi-criteria analysis for groundwater potential assessment in a structurally complex hard-rock terrain. Unlike conventional studies where resistivity data are primarily used qualitatively, the incorporation of transverse resistance and longitudinal conductance in this study enables a more quantitative evaluation of aquifer transmissivity and protective capacity, thereby improving the hydrogeological relevance of the zonation output. Furthermore, the results highlight the geological specificity of the Eastern Ghats Mobile Belt, where groundwater occurrence is strongly governed by fracture-controlled permeability and weathered mantle thickness, rather than primary porosity. The observed discrepancies between transmissivity values derived from Dar–Zarrouk parameters and those obtained from pumping tests highlight the critical role of fracture connectivity in enhancing groundwater yield in charnockitic and gneissic terrains. The validation of VES interpretations through field-based pumping test data and ROC analysis (AUC = 0.86) provides robust evidence of the model’s predictive capability, distinguishing this study from earlier works that rely solely on spatial overlay techniques. Thus, the study not only reinforces the applicability of integrated geophysical–geospatial approaches but also refines their use in complex crystalline terrains by emphasizing the need for combined quantitative parameterization and field validation.

The lithological interpretation based on resistivity data is in agreement with the observed hydrogeologic conditions and validates the use of the selected resistivity intervals and curve-type classification as a sound basis for aquifer delineation in the study area.

### Limitation of result

The present study has certain limitations that should be considered when interpreting the results. The geophysical investigation was conducted using single-season VES data, which may not fully capture seasonal variations in groundwater conditions and subsurface moisture distribution. In addition, the density of VES points is relatively limited, as only fifteen sounding locations were available to represent the hydrogeological variability across the study area. Although the VES locations were selected based on elevation and spatial distribution shown in the location map, a higher density of sounding points combined with multi-season data would further improve the accuracy and reliability of subsurface interpretation and groundwater potential assessment.

## Conclusion

This study demonstrates the efficacy of integrating Vertical Electrical Sounding (VES) with geospatial analysis and multi-criteria decision-making for delineating groundwater potential in the geologically complex hard-rock terrain of the Jeypore Block, Odisha. The integration of 15 VES surveys with 12 thematic layers using the Analytical Hierarchy Process (AHP) enabled systematic classification of the 456 km² area into distinct groundwater potential zones: weak (0.67%), moderate (48.75%), strong (49.95%), and very strong (0.64%).

Key findings reveal that deeper geological sequences (> 190 m cumulative thickness) are concentrated in the southern and eastern regions, corresponding to zones of enhanced groundwater potential. HA-type resistivity curves at two locations (VES-4 and VES-14) identified saturated, conductive layers favorable for aquifer development, while the predominance of AAA-type curves elsewhere indicates groundwater occurrence restricted to shallow weathered zones above compact crystalline basement. The spatial coincidence of high groundwater potential with elevated lineament density (1.602–2.37 km/km²) and weathered charnockite occurrences confirms the fundamental control of structural fabrics and lithology on groundwater storage and movement.

This study combined field geophysics with the GIS-based multi-criteria analysis, which performed well. The ROC analysis resulted in an AUC of 0.86, with the results matching global studies conducted in similar hard rock hydrogeology environments. The pumping tests were another step in validating the results, which indicated that the fractured zones contain more water than indicated by the Dar-Zarrouk method. It emphasizes the importance of fracture characterization in hard rock aquifers.

This study provides a good foundation for the management of water resources in the Jeypore Block, supported by scientific evidence. It can be used to determine the optimal locations for the placement of bore wells with more precision. The method can be used in other hard rock environments across the globe, especially in environments with little data availability. In the future, it can be taken further with the inclusion of multi-season VES investigations, denser sounding grids, and sophisticated techniques like Electrical Resistivity Tomography (ERT)^65–68^.

### Key findings

1. Subsurface variability: Deeper and thicker geological layers were concentrated in the southern and eastern regions, indicating zones of higher groundwater potential.
2. Resistivity interpretation: HA-type curves marked saturated zones favorable for aquifer development, while AAA-type curves indicated compact and less permeable basement rocks.
3. Visualization tools: Software such as Surfer-25, RockWorks-17, and IPI2WIN provided precise modeling and enhanced interpretation of subsurface features.
4. Multi-criteria analysis: The AHP method within GIS enabled the integration of slope, geology, drainage, and land use data to delineate groundwater potential zones.
5. Geological influence: Fractured and weathered charnockite in hilly terrains supported groundwater recharge, underlining the role of bedrock structure.
6. Methodological robustness: The approach proved effective and reproducible, validating its use through consistency with global studies in similar hydrogeological contexts.

## Supplementary Information

Below is the link to the electronic supplementary material.

Supplementary Material 1

## Data Availability

Data will be made available on a request basis.
