# Supplementary material for: Assessment of hydrogeophysical delineation of groundwater zones using vertical electrical sounding (VES) in Jeypore Block, Koraput District, Odisha
Source: Sci Rep. 2026 Jul 27;16:23336. doi: 10.1038/s41598-026-59721-2 (PMC13408355; doi:10.1038/s41598-026-59721-2)
Supplement: Supplementary file 1 — Supplementary Material 1 [file 41598_2026_59721_MOESM1_ESM.docx]

**Methodology**

**(a). Geo-electrical Sounding**

To assess subsurface geological formations and groundwater potential, Vertical Electrical Sounding (VES) was performed using the ABEM Terrameter SAS1000, a precision resistivity instrument with a built-in signal averaging system. The Schlumberger array configuration was employed to achieve deeper penetration and improved resolution, with a maximum electrode spread of 800 meters—400 meters on either side of the central point—consistent with methodologies used by Jimoh et al. (2023) and Ojo et al. (2024). Ten VES points were systematically distributed in a grid pattern across the study area, though adjustments were made to account for terrain constraints such as gullies, depressions, and existing infrastructure. Data acquisition was conducted under stable weather conditions, with electrode spacing progressively increased to probe various depths, and resistance values calculated from measured voltage drops and applied current, in line with procedures outlined by Wang et al. (2023) (**Eq. 1)**.

$\boldsymbol{R=}\frac{\boldsymbol{V}}{\boldsymbol{I}}$ **Eq. 1**

1. The apparent resistivity (ρₐ) for each station was computed by applying a geometric factor (K) specific to the Schlumberger array, using the formula (**Eq. 2**):

$\boldsymbol{K=\pi(}\frac{\boldsymbol{a}^{\boldsymbol{2}}\boldsymbol{-}\boldsymbol{b}^{\boldsymbol{2}}}{\boldsymbol{b}}\boldsymbol{)}$ **and** $\boldsymbol{\rho}_{\boldsymbol{a}}\boldsymbol{=K\times R}$ **Eq. 2**

Here, a denotes the half-spacing between current electrodes, and b the spacing between potential electrodes. The iterative measurements and corresponding calculations yielded accurate resistivity values, crucial for distinguishing lithological boundaries and detecting potential aquifer layers beneath the surface (Li et al., 2024; Ojo et al.,2024).

**(b). Data processing**

The VES 3.0 software was used to analyse the apparent resistivity and electrode spacing (AB/2) data collected during the study, allowing for efficient and precise modeling of subsurface conditions (George et al.,2025). Through multiple iterations, the software minimized error and optimized the match between observed and calculated curves, accurately estimating layer resistivities, thicknesses, and depths (Ibrahim et al., 2023). This iterative modeling process was crucial in delineating geological formations and evaluating their hydrogeological significance, ultimately supporting informed groundwater resource management (Sarkar et al.,2024).

**(c). The Dar-Zarrouk Parameters**

The evaluation of subsurface aquifer systems relies not only on qualitative interpretation of resistivity curves but also on quantitative derivations. Among these, the **Dar-Zarrouk parameters**—originating from layered resistivity models—are pivotal for understanding the groundwater transmission potential and the protective nature of overburden materials (**Bello et al., 2019**). These parameters, derived from **Vertical Electrical Sounding (VES)** data, offer critical insights into aquifer behavior, especially in areas where borehole data is sparse or unavailable.

**1. Total Layer Thickness (H)**

The total geoelectric thickness of the investigated profile is calculated as (**Eq. 3**):

**Eq. 3**

$$H=\sum_{i=1}^{n} h_{i}$$

Where:

*hi*_ihi​ is the thickness of the *i*th subsurface layer, *n* is the total number of geoelectric layers

This provides a cumulative measure of the depth profile under investigation.

**2. Longitudinal Conductance (S)**

The **longitudinal conductance** is a critical parameter used to assess the **protective capacity** of surface layers, particularly in clay-rich zones known for their low permeability and contaminant filtration ability (**Eq. 4**):

$$S=\sum_{i=1}^{n} \frac{h_{i}}{\rho_{i}}$$

**Eq. 4**

Where:

$\rho_{i}$​ is the true resistivity of the *i*th layer, $h_{i}$ *​* is the thickness of that layer.

Higher values of SSS indicate **better protective capacity** against pollutant infiltration, which is vital in groundwater contamination risk assessments.

**3.Transverse Resistances (T)**

In contrast, the **transverse resistance** is an indicator of a layer's ability to **transmit groundwater**, particularly within aquifer zones dominated by sand, gravel, or fractured rock (**Eq. 5**):

**Eq. 5**

$$T=\sum_{i=1}^{n} h_{i}\times\rho_{i}$$

Larger values of T suggest **greater transmissivity** and are often associated with highly permeable formations, aiding in the identification of productive groundwater zones.

**4. Longitudinal and Transverse Resistivity**

These parameters aim to describe **average resistivity behavior** through vertically stratified layers—useful when the subsurface is **anisotropic** (properties differ in different directions) (Eq.6).

1. Longitudinal Resistivity:

$$R_{s}=\frac{\boldsymbol{H}}{\boldsymbol{S}}$$

**Eq. 6**

H=$\sum h_{i}$ total thickness of the layered section. S= $\sum\frac{h_{i}}{\rho_{i}}$ Longitudinal conductance. Lower ​ $R_{s}$often indicates more conductive (clayey) or water-saturated zones. Represents **average resistivity** along the **direction of current flow (**Eq. 7**).**

1. Transverse Resistivity:

**Eq. 7**

$$R_{t}=\frac{\boldsymbol{T}}{\boldsymbol{H}}$$

T=$\sum h_{i}\rho_{i}$ transverse resistance, H=$\sum h_{i}$ total thickness. Represents **average resistivity perpendicular** to the current flow (horizontal). **Higher** $R_{t}$​ values usually mean more resistive layers—like dry sands, gravels, or fractured rock.

5. Aquifer Transmissivity (Tᵣ)

In hydrogeology, **transmissivity (Tᵣ)** defines the capability of an aquifer to transmit water under hydraulic gradients. It is given by (Eq. 8):

**Eq. 8**

$$\boldsymbol{T}_{\boldsymbol{r}}=\boldsymbol{k}\times\boldsymbol{h}$$

Where: k is the hydraulic conductivity (m/day). h is the thickness of the aquifer (m). High ​$\boldsymbol{T}_{\boldsymbol{r}}$ → the aquifer can deliver large volumes of water quickly, which is essential for borehole yield, irrigation, and water supply. Low $\boldsymbol{T}_{\boldsymbol{r}}$​ → water moves slowly, even if the aquifer is thick.

**(ii). Remote Sensing and GIS techniques.**

To examine spatial and temporal patterns within the study area, elevation data sourced from the SRTM mission and DEMs were utilized. A consistent flow of satellite imagery, captured every 16 days under the Global System-2 framework, supported long-term observation and analysis. The integration of multispectral data from the Landsat 8 OLI and TIRS sensors, with their advanced thermal and optical capabilities, greatly improved the detection of groundwater-related features. High-resolution datasets, including thermal bands, were merged to enhance the precision of environmental mapping. Using ArcGIS 10.7, multiple thematic layers were developed and overlaid to identify zones with high groundwater potential. The choice of datasets was strategically aligned with research goals and constrained by data accessibility.

**(a). Geospatial Data acquisition and processing**

Landsat 8 images were downloaded from the USGS Earth Explorer website. The images were used to create a land cover map. ArcGIS 10.7 software helped with this process. ArcGIS 10.7 was also used to make topographic and drainage maps. These maps were created using a Digital Elevation Model (DEM). Radiometric and geometric corrections were applied to the satellite images. Calibration and DOS tools were also used to improve the image quality. The final land cover map has five different classes (Fig. iso-resistivity).

Surfer 25 software was used to draw iso-resistivity maps. Rockworks 17 was used to create lithological bar graphs and fence diagrams of the subsurface.

**(b). Layering**

The geology of the region significantly influences groundwater occurrence, movement, infiltration, and runoff processes (Appukuttan & Reghunath, 2022; Egbueri et al., 2025).

Geomorphological features also play a vital role in groundwater recharge and distribution, and for this study, these features were validated using reference raster topographic maps of the area (Chatterjee et al., 2023; Chatterjee et al., 2024).

Additionally, soil structure critically affects infiltration rates, with fine-grained soils typically exhibiting lower permeability and porosity, thereby reducing groundwater recharge potential (Das et al., 2019; Chatterjee et al., 2024).

Lineaments are linear geological and geomorphological features that often indicate tectonic deformation, and their density is crucial for mineral exploration, geothermal resource assessment, soil erosion studies, earthquake analysis, and identifying groundwater potential zones (Allafta et al., 2020). In this study, the lineament density was analyzed using ArcGIS 10.7 software with a grid cell method (Eq. 8), as outlined by Moharir et al. (2023).

$\mathbf{LD}\boldsymbol{=}\sum_{\boldsymbol{i=1}}^{\boldsymbol{i=n}} \left( \frac{\boldsymbol{Li}}{\boldsymbol{A}} \right)\boldsymbol{(km}^{\boldsymbol{-1}}\boldsymbol{)}$ **Eq. 8**

Where LD is the lineament density, Li is the sum of the length of all the lineaments (km), i represents each linear feature in the study area, and A is the effective area of lineament cell grids (in square kilometres).

In areas with high drainage density, infiltration rates are generally low, as drainage density is inversely related to infiltration capacity, while factors like topography, geomorphology, and land use/land cover (LULC) show a positive correlation with drainage density. The drainage density in the study area was determined using the equation (Eq. 9) as described by Moharir et al. (2023).

$\mathbf{D}_{\mathbf{d}}\mathbf{=}\mathbf{L}\mathbf{/}\mathbf{A}$ **Eq. 9**

Dd is the drainage density, L is the length of drainage, and A is per unit of area.

To establish a crucial element of the research methodology, rainfall data were obtained from the Rainfall Monitoring System of Odisha (Chatterjee et al., 2025). The annual rainfall in the region fluctuates between 1562 mm and 1635 mm. To calculate the annual average rainfall from the monthly rainfall data (**Eq. 10**), you sum up the rainfall values for each of the 12 months of the year and then divide by 12. Here's the formula:

$\boldsymbol{R}_{\boldsymbol{annual} \boldsymbol{avg}\boldsymbol{}}\boldsymbol{=}\frac{\boldsymbol{R}_{\boldsymbol{Jan}}\boldsymbol{+}\boldsymbol{R}_{\boldsymbol{Feb}}\boldsymbol{+}\boldsymbol{R}_{\boldsymbol{Mar}}\boldsymbol{+\ldots+}\boldsymbol{R}_{\boldsymbol{Dec}}}{\boldsymbol{2}\boldsymbol{a}}$ **Eq. 10**

Where, $\boldsymbol{R}_{\boldsymbol{Jan}}\boldsymbol{+}\boldsymbol{R}_{\boldsymbol{Feb}}\boldsymbol{+}\boldsymbol{R}_{\boldsymbol{Mar}}\boldsymbol{+\ldots+}\boldsymbol{R}_{\boldsymbol{Dec}}$ = Monthly rainfall values (in mm or another unit), $\boldsymbol{R}_{\boldsymbol{annual avg}\boldsymbol{}}$= Annual average rainfall (in mm or the same unit as the monthly values).

Slope plays a significant role in influencing surface water infiltration, as well as the occurrence and storage of groundwater (Li et al., 2023). In this study, slope data were obtained from the SRTM DEM. The formula of Slope classification on Degree unit is given below (**Eq.11**):

$\mathbf{Slope}\mathbf{(}\boldsymbol{\theta}\mathbf{)}\mathbf{=}\mathbf{arctan}\frac{\sqrt{\mathbf{(}\mathbf{Z}_{\mathbf{x}}\mathbf{)}^{\mathbf{2}}\mathbf{+(}\mathbf{Z}_{\mathbf{y}}\mathbf{)}^{\mathbf{2}}}}{\mathbf{1}}$ **Eq.11**

Where, $\mathbf{Z}_{\mathbf{x}}$​ = Slope in the **x-direction** (east-west direction), $\mathbf{Z}_{\mathbf{y}}$ = Slope in the **y-direction** (north-south direction), arctan = The inverse tangent function (also called **atan**), θ = Slope in degrees.

Topographical features, such as hilly and steep terrains, tend to increase runoff and reduce infiltration, while flat plains support water retention and facilitate higher groundwater recharge through enhanced infiltration (Mukherjee and Singh 2020). The elevation value Z at a specific location (grid cell) in the DEM is provided directly by the DEM itself. No complex formula is required to calculate the elevation at each grid point. The elevation at any point (i, j) on the DEM is typically expressed as (**Eq.12**):

$\mathbf{Z}_{\mathbf{i}\mathbf{,}\mathbf{j}}\mathbf{=}\mathbf{Elevation} \mathbf{value} \mathbf{at} \mathbf{cell}\mathbf{(}\mathbf{i}\mathbf{,}\mathbf{j}\mathbf{)}$ **Eq.12**

Where,​$\boldsymbol{Z}_{\boldsymbol{i,j}}$ is the elevation (altitude) value at grid cell (*i,j*).This is usually in meters, depending on the DEM’s dataset. The classification of topography can be done by analyzing elevation values, which are derived from the DEM. Different topographic features can be identified by applying thresholds to the elevation values.

Land use and land cover (LULC) in the study area were mapped using Enhanced Thematic Mapper Plus (ETM+) satellite imagery, with twelve classes identified through supervised classification using the Maximum Likelihood Classification (MLC) algorithm. This algorithm determines the probability of each pixel belonging to a specific class by applying the (**Eq. 13**) Statistical Multivariate Normal Cumulative Distribution Function (CDF), which utilizes the mean, variance, and covariance of training samples from the Landsat data (Shin et al., 2019; Chatterjee et al., 2025).

$\boldsymbol{P (}\frac{\boldsymbol{X}}{\boldsymbol{Wi}}\boldsymbol{)}\mathbf{=}\frac{\frac{\mathbf{1}}{\mathbf{n}}}{\mathbf{(}\frac{\frac{\boldsymbol{2\pi}}{\mathbf{Vi}}}{\mathbf{0.5}}\mathbf{)}}\mathbf{exp}\boldsymbol{[}{\boldsymbol{-0.5}\left( \boldsymbol{X-Mi} \right)}^{\boldsymbol{T}}\boldsymbol{Vi}^{\boldsymbol{-1}}\boldsymbol{(X-Mi)}$**] Eq. 13**

Where, ‘n’ = the number of multispectral bands, ‘X’ = unknown measurement vector, ‘Vi’ = covariance matrix for each training class, ‘M’ = mean vector of each training class. The MLC Model (Maximum Likelihood Classifications) associated with land use and land cover change indicates a gradual shift in the climate of the study region (Chatterjee et al.,2025).

The Normalized Difference Vegetation Index (NDVI), which uses red and near-infrared bands (Eq. 3), is a commonly used tool to evaluate vegetation cover. NDVI values (**Eq. 14**), ranging from -1 to +1, are closely linked to groundwater potential; values near +1 indicate dense vegetation and strong groundwater potential, while values near -1 correspond to deep water bodies (Chatterjee et al.,2025).

$\boldsymbol{NDVI=}\frac{\boldsymbol{(NIR-RED)}}{\left( \boldsymbol{NIR+RED} \right)}$ **Eq. 14**

In this study, NDVI values span from -0.38 to +0.75, with regions exhibiting higher vegetation quality typically linked to shallow groundwater levels, making higher NDVI values more significant in the analysis (Chatterjee et al.,2025).

Groundwater fluctuation rates play a crucial role in defining groundwater priority zones, with levels generally reaching their lowest point before the monsoon season (March and May) and rising after the monsoon (October and December) due to the replenishment of water through percolation, with the fluctuation rate commonly calculated based on changes in groundwater levels over time (***Eq.15***), as expressed in the following formula (Li et al., 2021):

$Groundwater Fluctuation Rate (GFR\boldsymbol{)=}\frac{\boldsymbol{H}_{\boldsymbol{final}}\boldsymbol{-}\boldsymbol{H}_{\boldsymbol{initial}}}{\boldsymbol{ߡ}\boldsymbol{t}}$ ***Eq.15***

Where, $\boldsymbol{H}_{\boldsymbol{final}}$​ = Final groundwater level (at the end of the period), $\boldsymbol{H}_{\boldsymbol{initial}}$​ = Initial groundwater level (at the start of the period), *Δt* = Time period of fluctuation (in months or years). This formula can be applied to estimate the fluctuation rate over specific periods, such as between pre-monsoon and post-monsoon seasons.

The groundwater table level (Wt) and aquifer thickness (At) are key hydrogeological factors that determine the quantity and accessibility of groundwater in a region (Li et al., 2021). Hydrogeological mapping for this study was carried out using data from the Central Ground Water Board (CGWB) and the Geological Survey of India (GSI). o quantifies the relationship between the groundwater table level (Wt) and aquifer thickness (At), a general formula for groundwater volume or storage can be expressed as (**Eq.16**):

$\boldsymbol{V}_{\boldsymbol{GW}}\mathbf{=}\boldsymbol{A}\boldsymbol{\times}\boldsymbol{A}_{\boldsymbol{t}}$**×**$\boldsymbol{W}_{\boldsymbol{t}}$ **Eq.16**

Where, VGW​ = Groundwater volume or storage (in cubic meters), A = Area of the study region (in square meters), At​ = Aquifer thickness (in meters), Wt​ = Groundwater table level or depth (in meters). This formula provides an estimate of the groundwater volume in a given region, which is influenced by both the thickness of the aquifer and the depth to the groundwater table.

(c). **Analytical Hierarchy Processes**

The Analytical Hierarchical Process (AHP) is a widely adopted GIS-based technique for identifying potential groundwater zones (Mukherjee & Singh, 2020). In this study, nine key parameters were selected, and a pairwise comparison matrix was constructed to assign proportional weights to each class and normalize them for calculating the cumulative influence of the main criteria (Kumar et al., 2024; Dar et al., 2021). The consistency ratio (CR) was computed to ensure reliability, and all thematic layers were integrated based on their relative influence on groundwater potential using the AHP methodology (Dandapat et al., 2024).

**Pairwise comparison of criteria**

The first step in the criteria comparison involved conducting pairwise comparisons, with the results subsequently entered into a comparison matrix. Following the approach outlined by (Saaty, 1980; Dar et al.,2021; Mukherjee and Singh, 2020), each criterion was evaluated against the others based on its relative importance, using a scale from 1 to 9 (Table 2).

Table 2. Pairwise comparison scale

| **Sl. No.** | **Scale description AHP value** | **AHP integer value** |
| --- | --- | --- |
| **1** | Equally important | 1 |
| **2** | Equally to moderately important | 2 |
| **3** | Moderately important | 3 |
| **4** | Moderately to strongly important | 4 |
| **5** | Strongly important | 5 |
| **6** | Strongly to very strongly important | 6 |
| **7** | Very strongly important | 7 |
| **8** | Very strongly to extremely important | 8 |
| **9** | Extremely important | 9 |

**Calculating criterion weights**

To manually calculate the criterion weights, the following steps were performed (Saaty, 1980; Dar et al.,2021; Mukherjee and Singh, 2020):

The values in each column of the **pairwise comparison matrix** were summed;

Pairwise comparison matrix (PCM) calculated using equation 17.

$\boldsymbol{A}\boldsymbol{1=}\left[ \begin{matrix} \begin{matrix} {\boldsymbol{a´}_{\boldsymbol{11}}\boldsymbol{a´}}_{\boldsymbol{12}}\boldsymbol{\cdots} \\ {\boldsymbol{a´}_{\boldsymbol{21}}\boldsymbol{a´}}_{\boldsymbol{22}}\boldsymbol{\cdots} \\ \boldsymbol{\vdots\vdots\ddots} \end{matrix} & \begin{matrix} \boldsymbol{a´}_{\boldsymbol{1}\boldsymbol{n}} \\ \boldsymbol{a´}_{\boldsymbol{2}\boldsymbol{n}} \\ \boldsymbol{\vdots} \end{matrix} \\ \boldsymbol{a´}_{\boldsymbol{n}\boldsymbol{1}} \boldsymbol{a´}_{\boldsymbol{n}\boldsymbol{2}}\boldsymbol{\cdots} & \boldsymbol{a´}_{\boldsymbol{nn}} \end{matrix} \right]\boldsymbol{,A=}\frac{\boldsymbol{a}_{\boldsymbol{ij}}}{\sum_{\boldsymbol{1}}^{\boldsymbol{n}} \boldsymbol{a}_{\boldsymbol{ij}}}\boldsymbol{f or i,j}\boldsymbol{= 1, 2, \ldots,}\boldsymbol{n}$ **Eq. 17**

Where A1 is the Pairwise comparison matrix, **a**_nn_ is the indicator of the Pairwise matrix element.

Next, to solve the reciprocal matrix and measure the relative value of parameters, an eigenvector technique is used (**Eq. 18 & 19).**

$\boldsymbol{W=}\left[ \begin{aligned} \boldsymbol{W}_{\boldsymbol{1}} \\ \boldsymbol{W}_{\boldsymbol{2}} \\ \boldsymbol{\vdots} \\ \boldsymbol{W}_{\boldsymbol{n}} \end{aligned} \right]\boldsymbol{and Wi=}\frac{\sum_{\boldsymbol{1}}^{\boldsymbol{n}} \boldsymbol{a}_{\boldsymbol{ij}}}{\boldsymbol{n}}\boldsymbol{for i = 1, 2 \ldots., n}\& \boldsymbol{W}^{\boldsymbol{'}}$ **Eq.18**

$\boldsymbol{AW=}\left[ \begin{aligned} \boldsymbol{w´}_{\boldsymbol{1}} \\ \boldsymbol{w´}_{\boldsymbol{2}} \\ \boldsymbol{\vdots} \\ \boldsymbol{w´}_{\boldsymbol{n}} \end{aligned} \right]\boldsymbol{,}\&\boldsymbol{\lambda}_{\boldsymbol{max}}\boldsymbol{=}\frac{\boldsymbol{1}}{\boldsymbol{n}}\boldsymbol{(}\frac{\boldsymbol{W}_{\boldsymbol{1}}^{\boldsymbol{´}}}{\boldsymbol{W}_{\boldsymbol{1}}^{\boldsymbol{´}}}\boldsymbol{+}\frac{\boldsymbol{W}_{\boldsymbol{1}}^{\boldsymbol{´}}}{\boldsymbol{W}_{\boldsymbol{2}}^{\boldsymbol{´}}}\boldsymbol{+\cdots+}\frac{\boldsymbol{W}_{\boldsymbol{n}}^{\boldsymbol{´}}}{\boldsymbol{W}_{\boldsymbol{n}}^{\boldsymbol{´}}}\boldsymbol{)}$ **Eq.19**

Where, W is the Eigen vector, Wi is the Eigen value of criterion i, and $\lambda_{max}$ is the average of the eigenvalue of the pair wise comparison matrix.

**Table 3** | Relative significance scale and Random consistency index (RCI) constructed on Saaty, 1980.

| Intensity  of significance | 1 | 2 | 3 | 4 | 5 | 6 | 7 | 8 | 9 | 10 | 11 | 12 |
| --- | --- | --- | --- | --- | --- | --- | --- | --- | --- | --- | --- | --- |
| Definition | Equal | Weak | Mode-rate | Mode-  rate  plus | Strong | Strong  plus | Very  strong | Very  Very  strong | Extreme | Very Extreme |  |  |
|  |  |  |  |  |  |  |  |  |  |  |  |  |
| No. of  parameters nominated | 1 | 2 | 3 | 4 | 5 | 6 | 7 | 8 | 9 | 10 | 11 | 12 |
| RCI value | 0 | 0 | 0.58 | 0.9 | 1.12 | 1.12 | 1.32 | 1.41 | 1.45 | 1.49 | 1.51 | 1.48 |

Each matrix element was divided by its respective column sum (**Eq.20**), resulting in the **normalized pairwise comparison matrix**; For each element $a_{ij}${ij}aij​ in the matrix $A_{ˎ}$ the normalized value $\hat{a_{ij}}$​ is calculated as:

${\hat{\boldsymbol{a}}}_{\boldsymbol{ij}}$**=**$\frac{\boldsymbol{a}_{\boldsymbol{ij}}}{\boldsymbol{S}_{\boldsymbol{j}}}$ **Eq.20**

Where, ${\hat{\boldsymbol{a}}}_{\boldsymbol{ij}}$is the normalized value of the element in the iii-th row and jjj-th column, $\boldsymbol{a}_{\boldsymbol{ij}}$is the original element in the iii-th row and jjj-th column, $\boldsymbol{S}_{\boldsymbol{j}}$is the sum of the values in column j.

**The sum of the normalized values for each row was multiplied by the total number of criteria to determine the average of the elements in each row.** For this study, AHP software was employed to compute the final criterion weights ($\boldsymbol{Eq.21}$). The formula to determine the average of the elements in each row is as follows:

$$\boldsymbol{\omega}_{\boldsymbol{i}}\frac{\boldsymbol{1}}{\boldsymbol{n}}\sum_{\boldsymbol{J}\boldsymbol{=}\boldsymbol{1}}^{\boldsymbol{n}} {\hat{\boldsymbol{a}}}_{\boldsymbol{ij}} \boldsymbol{Eq}\boldsymbol{.}\boldsymbol{21}$$

Where, $\boldsymbol{\omega}_{\boldsymbol{i}}$ is the weight of the i-th criterion, ${\hat{\boldsymbol{a}}}_{\boldsymbol{ij}}$ is the normalized value in the i-th row and jjj-th column of the normalized pairwise comparison matrix $A_{ˎ}$.

**Table 4|** Pair-wise comparison matrix of twelve influencing factors and their normalized weight of the Groundwater priority zone.

| **Factors** | **GEO** | **GEOM** | **SOI** | **NDVI** | **LD** | **DD** | **RF** | **SLP** | **PHY** | **LULC** | **GWF** | **HYDG** | **Geometric mean** | **Normalized weight** |
| --- | --- | --- | --- | --- | --- | --- | --- | --- | --- | --- | --- | --- | --- | --- |
| GEO | **1.00** | **1.09** | **1.20** | **1.33** | **1.50** | **1.71** | **2.00** | **2.40** | **3.00** | **4.00** | **6.00** | **12.00** | **3.10321** | **0.203** |
| GEOM | **0.92** | **1.00** | **1.00** | **1.00** | **1.10** | **1.22** | **1.38** | **1.57** | **1.83** | **2.20** | **2.75** | **3.67** | **1.63628** | **0.107** |
| SOI | **0.83** | **1.00** | **1.00** | **1.00** | **1.10** | **1.22** | **1.38** | **1.57** | **1.83** | **2.20** | **2.75** | **3.67** | **1.62933** | **0.107** |
| NDVI | **0.75** | **1.00** | **1.00** | **1.00** | **1.10** | **1.22** | **1.38** | **1.57** | **1.83** | **2.20** | **2.75** | **3.67** | **1.62239** | **0.106** |
| LD | **0.67** | **0.91** | **0.91** | **0.91** | **1.00** | **1.00** | **1.13** | **1.29** | **1.50** | **1.80** | **2.25** | **3.00** | **1.36289** | **0.089** |
| DD | **0.58** | **0.82** | **0.82** | **0.82** | **1.00** | **1.00** | **1.13** | **1.29** | **1.50** | **1.80** | **2.25** | **3.00** | **1.33322** | **0.087** |
| RF | **0.50** | **0.73** | **0.73** | **0.73** | **0.89** | **0.89** | **1.00** | **1.00** | **1.00** | **1.14** | **1.33** | **1.60** | **0.96132** | **0.063** |
| SLP | **0.42** | **0.64** | **0.64** | **0.64** | **0.78** | **0.78** | **1.00** | **1.00** | **1.00** | **1.14** | **1.33** | **1.60** | **0.91313** | **0.060** |
| PHY | **0.33** | **0.55** | **0.55** | **0.55** | **0.67** | **0.67** | **1.00** | **1.00** | **1.00** | **1.14** | **1.33** | **1.60** | **0.86494** | **0.057** |
| LULC | **0.25** | **0.45** | **0.45** | **0.45** | **0.56** | **0.56** | **0.88** | **0.88** | **0.88** | **1.00** | **1.17** | **1.40** | **0.74303** | **0.049** |
| GWF | **0.17** | **0.36** | **0.36** | **0.36** | **0.44** | **0.44** | **0.75** | **0.75** | **0.75** | **0.86** | **1.00** | **1.00** | **0.60447** | **0.040** |
| HYDG | **0.08** | **0.27** | **0.27** | **0.27** | **0.33** | **0.33** | **0.63** | **0.63** | **0.63** | **0.71** | **1.00** | **1.00** | **0.51309** | **0.034** |
| **SUM** | **6.50** | **8.82** | **8.93** | **9.06** | **10.47** | **11.05** | **13.63** | **14.94** | **16.75** | **20.20** | **25.92** | **37.20** | **15.287** | **1.00** |

(**GEO**: Geology, **GEOM**: Geomorphology, **SOI**: Soil, **NDVI**: Normalized Differenc, Vegetation Index, **LD**: Lineament Density, **DD**: Drainage Density, **RF**: Rainfall, **SLP**: Slope, **PHY**: Physiography, **LULC**: Land Use/Land Cover, **GWF**: Groundwater Fluctuation, **HYDG**: Hydrogeology).

**Calculation of principal eigenvalue vector**

To calculate the **principal eigenvalue vector** (also known as the weight vector in AHP), we solve the **eigenvalue problem** for the pairwise (**Eq.22**) comparison matrix A (Saaty, 1980; Dar et al.,2021; Mukherjee and Singh, 2020). The general formula is:

1. $\boldsymbol{A.\omega=}\boldsymbol{\lambda}_{\boldsymbol{Max}}\boldsymbol{.\omega}$ **Eq.22**

Where, $\boldsymbol{A}$ is the pairwise comparison matrix (*size n×n*), $\boldsymbol{\omega}$ is the eigenvector (the principal eigenvalue vector.$\boldsymbol{\lambda}_{\boldsymbol{Max}}$ is the principal eigenvalue (the largest eigenvalue of the matrix),n is the number of criteria (or size of the matrix).

This equation expresses that when you multiply the matrix A by its eigenvector $\boldsymbol{\omega}$, You get a scalar multiple of the eigenvector. The scalar $\boldsymbol{\lambda}_{\boldsymbol{Max}}$​ is the **principal eigenvalue**.

**Table 5|** Calculation of principal eigenvalue table (λ_max_) of Groundwater Priority Zone.

| **Parameters** | **Sum of the Column** | **Weight vector** | **Parameter rank (A×B)** |
| --- | --- | --- | --- |
| **GEO** | **7.42** | **0.203** | **1.5062** |
| **GEOM** | **7.42** | **0.107** | **0.7942** |
| **SOI** | **8.91** | **0.107** | **0.9496** |
| **NDVI** | **9.13** | **0.106** | **0.9689** |
| **LD** | **10.77** | **0.089** | **0.9602** |
| **DD** | **11.34** | **0.087** | **0.9890** |
| **RF** | **14.29** | **0.063** | **0.8986** |
| **SLP** | **15.53** | **0.060** | **0.9276** |
| **PHY** | **18.43** | **0.057** | **1.0427** |
| **LULC** | **21.83** | **0.049** | **1.0610** |
| **GWF** | **27.14** | **0.040** | **1.0731** |
| **HYDG** | **36.33** | **0.034** | **1.2194** |
|  | | | **12.3906** |

**Calculating consistency ratio (CR)**

The **Consistency Ratio (CR)** is a measure used in the **Analytic Hierarchy Process (AHP)** to evaluate how consistent the judgments are in the pairwise comparison matrix. A lower CR indicates that the judgments are relatively consistent, while a higher CR suggests that the judgments may be inconsistent and need to be revised.

To calculate the **Consistency Ratio (CR)**, you need the **Consistency Index (CI)** and the **Random Consistency Index (RI)**.

The Consistency ratio (CR) is used to validate the AHP judgment matrix using the equation below (**Eq. 23**) (Saaty, 1980; Dar et al.,2021; Mukherjee and Singh, 2020)

1. $\mathbf{CR=}\frac{\mathbf{CI}}{\mathbf{RI}}$ **Eq. 23**

Where CR is calculated by dividing CI (Consistency index) by RI (Random consistency index) using Saaty’s equation (1980) and Dar et al.,2021; Mukherjee and Singh, 2020.

1. $\mathbf{CI=}\frac{\boldsymbol{(\lambda max-n)}}{\mathbf{(n-1)}}$ **Eq.24**

Where CI is the consistency index, and λmax denotes the eigenvalue of the X matrix, n is the number of input indicators respectively (**Eq. 24**), which are illustrated in **Table 5**. CR value obtained is 2.3992 %, which is less than 10%, thus justifying the consistency in the pairwise comparison (Saaty, 1980; Dar et al.,2021; Mukherjee and Singh 2020).

1. $CI\%=\frac{CI}{RI}X 100=2.36\%$**Eq. 25**

In this study, the number of factors (n) = 12, where RI = 1.48 (**Table 2)** and CR = 0.03< 0.1, when CR is less than 10%, it is acceptable. However, the value of CR in the research is less than 10%, so it is important enough to continue the analysis (**Eq. 25**).

**Table 6 |** Consistency Ratio and Consistency Index Table

| **λmax** | **n** | **λmax-n** | **n-1** | **CI** | **RI** | **CI/ RI** | **CR** | **PERCENT** | **CR%** |
| --- | --- | --- | --- | --- | --- | --- | --- | --- | --- |
| **12.3906** | **12** | **0.3906** | **11** | **0.035** | **1.48** | **0.0236** | **0.0236** | **100** | **2.36** |

The features within each theme were assigned and normalized weights using techniques like the Analytical Hierarchy Process (AHP) to determine their relative importance in groundwater delineation (Table 7).

| Sl. No. | VES No | Place of Village | Latitude | longitude | Layer | Layer resistivity Ohm/m | Resistivity Meter | Layer thickness in meter | Theckness Meter | Communicative thickness (Depth in Meters) | Depth in Meters | Geological formation | Types of Curve | Lithological Intrepratation |
| --- | --- | --- | --- | --- | --- | --- | --- | --- | --- | --- | --- | --- | --- | --- |
|  |  |  |  |  |  |  |  |  |  |  |  |  |  |  |
| 1 | VES 1 | Singhibandha | 18° 58' 1.667" | 82° 39' 1.454" | 1 | P1 | 3.25 | h1 | 1.9 | D1 | 0.9 | Eastern Ghat super group hard rock | AAA | Top soil |
|  |  |  |  |  | 2 | P2 | 9.75 | h2 | 6.7 | D2 | 8.6 |  |  | Laterite |
|  |  |  |  |  | 3 | P3 | 53.5 | h3 | 28.3 | D3 | 36.9 |  |  | Granite |
|  |  |  |  |  | 4 | P4 | 121.5 |  |  |  |  |  |  | Bed rock |
| 2 | VES 2 | Kebidi | 18° 57' 4.679" | 82° 37' 31.901" | 1 | P1 | 1.83 | h1 | 1.2 | D1 | 1.2 | Eastern Ghat super group hard rock | AAA | Top soil |
|  |  |  |  |  | 2 | P2 | 8.25 | h2 | 4.2 | D2 | 5.4 |  |  | Laterite |
|  |  |  |  |  | 3 | P3 | 41.2 | h3 | 31.5 | D3 | 36.9 |  |  | Granite |
|  |  |  |  |  | 4 | P4 | 88.25 |  |  |  |  |  |  | Bed rock |
| 3 | VES 3 | Batojaganathpur | 18° 56' 59.794" | 82° 35' 45.523" | 1 | P1 | 2.9 | h1 | 1.5 | D1 | 1.5 | Eastern Ghat super group hard rock | AAA | Top soil |
|  |  |  |  |  | 2 | P2 | 6.7 | h2 | 2.5 | D2 | 4 |  |  | Sansstone & Laterite |
|  |  |  |  |  | 3 | P3 | 45.1 | h3 | 25.5 | D3 | 29.5 |  |  | Granite |
|  |  |  |  |  | 4 | P4 | 96.3 |  |  |  |  |  |  | Bed rock |
| 4 | VES 4 | Phupugam | 18° 59' 51.845" | 82° 34' 58.846" | 1 | P1 | 29.9 | h1 | 0.6 | D1 | 0.6 | Eastern Ghat super group hard rock | HA | Top soil |
|  |  |  |  |  | 2 | P2 | 2.6 | h2 | 7.4 | D2 | 8 |  |  | Laterite |
|  |  |  |  |  | 3 | P3 | 21.5 | h3 | 24.5 | D3 | 32.5 |  |  | Granite |
|  |  |  |  |  | 4 | P4 | 93.9 |  |  |  |  |  |  | Bed rock |
| 5 | VES 5 | Chikima | 19° 0' 10.205" | 82° 31' 51.468" | 1 | P1 | 14.4 | h1 | 3.98 | D1 | 3.98 | Eastern Ghat super group hard rock | AAA | Top soil |
|  |  |  |  |  | 2 | P2 | 58.8 | h2 | 7.12 | D2 | 11.1 |  |  | Sansstone & Laterite |
|  |  |  |  |  | 3 | P3 | 82.6 | h3 | 37.2 | D3 | 48.3 |  |  | Granite |
|  |  |  |  |  | 4 | P4 | 97.6 |  |  |  |  |  |  | Bed rock |
| 6 | VES 6 | Konga | 18° 59' 15.382" | 82° 28' 25.197" | 1 | P1 | 2 | h1 | 6.95 | D1 | 6.95 | Eastern Ghat super group hard rock | AAA | Top soil |
|  |  |  |  |  | 2 | P2 | 7 | h2 | 11.85 | D2 | 18.8 |  |  | Sansstone & Laterite |
|  |  |  |  |  | 3 | P3 | 45 | h3 | 41.5 | D3 | 60.3 |  |  | Granite |
|  |  |  |  |  | 4 | P4 | 91 |  |  |  |  |  |  | Bed rock |
| 7 | VES 7 | Kaliagam | 18° 54' 15.227" | 82° 29' 17.279" | 1 | P1 | 13 | h1 | 13.4 | D1 | 13.4 | Eastern Ghat super group hard rock | AAA | Top soil |
|  |  |  |  |  | 2 | P2 | 78.6 | h2 | 1.6 | D2 | 15 |  |  | Sansstone & Laterite |
|  |  |  |  |  | 3 | P3 | 92 | h3 | 16.4 | D3 | 31.4 |  |  | Granite |
|  |  |  |  |  | 4 | P4 | 119 |  |  |  |  |  |  | Bed rock |
| 8 | VES 8 | Kunturukhal | 18° 50' 33.092" | 82° 31' 33.933" | 1 | P1 | 16.4 | h1 | 16.4 | D1 | 16.4 | Eastern Ghat super group hard rock | AAA | Top soil |
|  |  |  |  |  | 2 | P2 | 76.6 | h2 | 2.9 | D2 | 19.3 |  |  | Sansstone & Laterite |
|  |  |  |  |  | 3 | P3 | 93 | h3 | 27 | D3 | 46.3 |  |  | Granite |
|  |  |  |  |  | 4 | P4 | 149 |  |  |  |  |  |  | Bed rock |
| 9 | VES 9 | Padmapur | 18° 49' 17.574" | 82° 34' 35.729" | 1 | P1 | 18.9 | h1 | 18.9 | D1 | 18.9 | Eastern Ghat super group hard rock | AAA | Top soil |
|  |  |  |  |  | 2 | P2 | 54.3 | h2 | 3.1 | D2 | 22 |  |  | Laterite |
|  |  |  |  |  | 3 | P3 | 77.1 | h3 | 29.5 | D3 | 51.5 |  |  | Granite |
|  |  |  |  |  | 4 | P4 | 127.7 |  |  |  |  |  |  | Bed rock |
| 10 | VES 10 | Mukhukhudupi | 18° 47' 1.062" | 82° 30' 38.727" | 1 | P1 | 16.1 | h1 | 13.4 | D1 | 13.4 | Eastern Ghat super group hard rock | AAA | Top soil |
|  |  |  |  |  | 2 | P2 | 61.5 | h2 | 4.2 | D2 | 17.6 |  |  | Laterite |
|  |  |  |  |  | 3 | P3 | 89.9 | h3 | 14.2 | D3 | 31.8 |  |  | Granite |
|  |  |  |  |  | 4 | P4 | 142.5 |  |  |  |  |  |  | Bed rock |
| 11 | VES 11 | Mohulobhota | 18° 53' 3.529" | 82° 37' 42.762" | 1 | P1 | 13.6 | h1 | 12.9 | D1 | 12.90 | Eastern Ghat super group hard rock | AAA | Top soil |
|  |  |  |  |  | 2 | P2 | 86.4 | h2 | 26.5 | D2 | 39.40 |  |  | Laterite |
|  |  |  |  |  | 3 | P3 | 100.3 | h3 | 11.5 | D3 | 50.90 |  |  | Granite |
|  |  |  |  |  | 4 | P4 | 119.7 | h4 | 26.1 | D4 | 77.00 |  |  | Bed rock |
| 12 | VES 12 | Dhonopur | 18° 56' 13.696" | 82° 31' 44.700" | 1 | P1 | 39.5 | h1 | 11.2 | D1 | 11.2 | Eastern Ghat super group hard rock | AAA | Top soil |
|  |  |  |  |  | 2 | P2 | 105.2 | h2 | 22.9 | D2 | 34.1 |  |  | Laterite |
|  |  |  |  |  | 3 | P3 | 205.5 | h3 | 36.8 | D3 | 70.9 |  |  | Granite |
|  |  |  |  |  | 4 | P4 | 409.5 | h4 | 22.5 | D4 | 93.4 |  |  | Bed rock |
| 13 | VES 13 | Putra | 18° 57' 44.496" | 82° 30' 15.613" | 1 | P1 | 63.5 | h1 | 8.3 | D1 | 8.3 | Eastern Ghat super group hard rock | AAA | Top soil |
|  |  |  |  |  | 2 | P2 | 82.5 | h2 | 32.5 | D2 | 40.8 |  |  | Sansstone & Laterite |
|  |  |  |  |  | 3 | P3 | 102.5 | h3 | 23.9 | D3 | 64.7 |  |  | Granite |
|  |  |  |  |  | 4 | P4 | 189.5 | h4 | 44.2 | D4 | 108.9 |  |  | Bed rock |
| 14 | VES 14 | Ekomba | 18° 54' 0.065" | 82° 32' 33.527" | 1 | P1 | 106.5 | h1 | 11.9 | D1 | 11.9 | Eastern Ghat super group hard rock | HA | Top soil |
|  |  |  |  |  | 2 | P2 | 79 | h2 | 29.5 | D2 | 41.4 |  |  | Laterite |
|  |  |  |  |  | 3 | P3 | 220 | h3 | 25.2 | D3 | 66.6 |  |  | Granite |
|  |  |  |  |  | 4 | P4 | 695 |  |  |  |  |  |  | Bed rock |
| 15 | VES 15 | Solopa | 18° 57' 8.776" | 82° 28' 6.437" | 1 | P1 | 30.4 | h1 | 20.4 | D1 | 20.4 | Eastern Ghat super group hard rock | AAA | Top soil |
|  |  |  |  |  | 2 | P2 | 95.6 | h2 | 73.6 | D2 | 94 |  |  | Sansstone & Laterite |
|  |  |  |  |  | 3 | P3 | 99.1 | h3 | 99.5 | D3 | 193.5 |  |  | Granite |
|  |  |  |  |  | 4 | P4 | 169.9 |  |  |  |  |  |  | Bed rock |
